# Theory of Excitons in Atomically Thin Semiconductors: Tight-Binding Approach

**DOI:** 10.3390/nano12091582

**Published:** 2022-05-06

**Authors:** Maciej Bieniek, Katarzyna Sadecka, Ludmiła Szulakowska, Paweł Hawrylak

**Affiliations:** 1 Department of Physics, University of Ottawa, Ottawa, ON K1N 6N5, Canada; katarzyna.sadecka@pwr.edu.pl (K.S.); ludziaa@gmail.com (L.S.); pawel.hawrylak@uottawa.ca (P.H.); 2 Department of Theoretical Physics, Wrocław University of Science and Technology, Wybrzeże Wyspiańskiego 27, 50-370 Wrocław, Poland; 3 Institut für Theoretische Physik und Astrophysik, Universität Würzburg, 97074 Würzburg, Germany

**Keywords:** tight-binding, excitons, Bethe–Salpeter equation, transition metal dichalcogenides

## Abstract

Atomically thin semiconductors from the transition metal dichalcogenide family are materials in which the optical response is dominated by strongly bound excitonic complexes. Here, we present a theory of excitons in two-dimensional semiconductors using a tight-binding model of the electronic structure. In the first part, we review extensive literature on 2D van der Waals materials, with particular focus on their optical response from both experimental and theoretical points of view. In the second part, we discuss our ab initio calculations of the electronic structure of MoS_2_, representative of a wide class of materials, and review our minimal tight-binding model, which reproduces low-energy physics around the Fermi level and, at the same time, allows for the understanding of their electronic structure. Next, we describe how electron-hole pair excitations from the mean-field-level ground state are constructed. The electron–electron interactions mix the electron-hole pair excitations, resulting in excitonic wave functions and energies obtained by solving the Bethe–Salpeter equation. This is enabled by the efficient computation of the Coulomb matrix elements optimized for two-dimensional crystals. Next, we discuss non-local screening in various geometries usually used in experiments. We conclude with a discussion of the fine structure and excited excitonic spectra. In particular, we discuss the effect of band nesting on the exciton fine structure; Coulomb interactions; and the topology of the wave functions, screening and dielectric environment. Finally, we follow by adding another layer and discuss excitons in heterostructures built from two-dimensional semiconductors.

## 1. General Overview

### 1.1. 2D van der Waals Materials

The fact that many crystals have a layered form with strong intralayer and weak interlayer bonds is well known [1,2,3]. Renewed widespread interest in such crystals reignited around 2005 due to the realization that high-quality few-layer crystals can be obtained via a mechanical exfoliation process [4,5,6]. Immediately, it was realized that other materials can be obtained using this technique [7], i.e., insulators, semiconductors, metals, and superconductors. More recently, large-scale data mining approaches established that from about $10^{5}$ experimentally known crystals, approximately 1800 should be exfoliable and thermodynamically stable [8]. Around 40 of them belong to the group called transition metal dichalcogenides (TMDs). In this family, contrary to graphene, a single layer is built from three planes of atoms: two of them are built out of chalcogen X, surrounding a symmetrically metal M plane (X-M-X structure). Usually, they have a trigonal (1T), hexagonal (2H), or rhombohedral (3R) polymorphic structure [9]. Around half of them in bulk form are metallic, and the other half are semiconducting [10], sharing the common theme that when thinned down, the electronic band gap increases. In several cases, the transition from metal to semiconductor is predicted. The semiconducting TMDs that sparked the greatest attention due to their promising optoelectronic properties [11] were compounds with formulas MoS${}_{2}$, MoSe${}_{2}$, MoTe${}_{2}$, WS${}_{2}$, and WSe${}_{2}$.

What is worth noting, is that the properties of the monolayer crystals depend not only on their chemical composition and structural phase, but also on the choice of the substrate. When the first samples of TMDs were obtained, the standard procedure after their exfoliation was to place them on a properly prepared SiO${}_{2}$/Si substrate, which for a certain thickness of the SiO${}_{2}$ layer enhanced the contrast in optical microscopy [12]. It was also understood that optical quality depends heavily on the surrounding dielectric environment [13,14], which posed the question as to whether SiO${}_{2}$ is truly the best substrate for optical studies. Currently, it is clear that the substrate on which the subtle optical features of TMDs can be most easily observed is hBN [15,16], for which, e.g., exciton lines approach ∼2 meV widths.

### 1.2. Optical Properties of TMDs and Their Heterostructures

One of the results that sparked widespread attention was the observation that MoS${}_{2}$, when thinned down to a single layer, exhibits significantly stronger photoluminescence (PL) than n≥2-layer crystals [17,18,19]. A similar trend was then confirmed in MoSe${}_{2}$ [20], WS${}_{2}$ [21], WSe${}_{2}$ [20,21], and MoTe${}_{2}$ [22,23] semiconductors with varying magnitudes of differences between samples and with different numbers of layers. This phenomenon was related to the indirect–direct band gap transition, caused by different renormalizations of indirect (Γ−Q) band gaps compared to the much smaller renormalizations of the K−K direct gap when samples are thinned down. This trend was confirmed theoretically by ab initio calculations [24,25] and experimentally by photoemission spectroscopy for valence bands [26].

**Figure 1:**
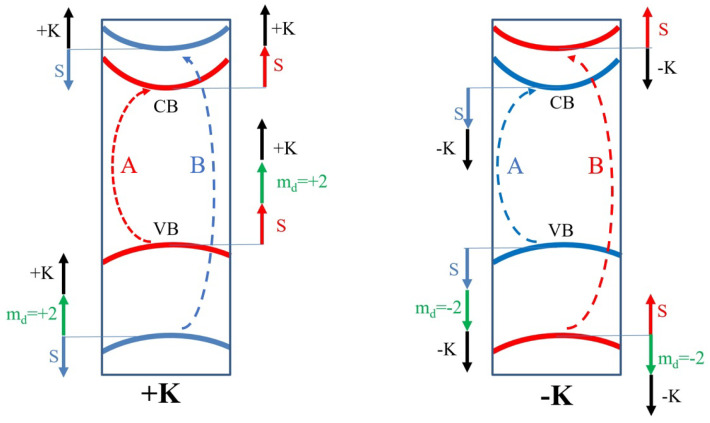
Schematic structure of spin-split bands in monolayer MX${}_{2}$+K/−K valleys. Respective A/B excitonic transitions are shown as dashed arrows. Additional solid arrows denote different contributions to the +K and −K valleys’ responses to magnetic fields. The solid blue and red arrows show the bare electron Zeeman contribution in magnetic field $\vec{B}=\left(0,0,B_{z}\right)$; the green—the atomic orbital Landé contribution and the black ones—the valley Zeeman contribution.

The first inkling that strong PL is dominated by an excitonic (X) transition comes from the comparison of the PL peak energy position to the known spectral positions of the excitonic transitions in bulk MoS${}_{2}$ at *K* point [17,27]. The generation of excitons in a given valley is possible via specific selection rules, which couple circularly polarized light with excitation in a given valley [28,29,30,31,32,33,34,35], (see Figure 1 for schematic picture). The valley coherence of such excitons has been probed in many experiments [36,37,38,39,40]. The spontaneous choice of valley after excitation with unpolarized light has also been observed [41], pointing towards a valley-polarized ground state [42,43,44]. Excited exciton states were measured in WSe${}_{2}$ [45,46] and WS${}_{2}$ [47] prior to molybdenum-based TMDs due to the larger separation between A and B excitons (∼0.4 eV), so that the B exciton was not “masking” the excited A exciton states. Complementary PL excitation spectroscopy allowed for the measurement of excited states in MoS${}_{2}$, MoSe${}_{2}$, WS${}_{2}$, and WSe${}_{2}$ [48,49,50]. The excited B exciton series is more challenging to measure [46,49] due to overlap with the wide PL signal from so-called C excitons. All of those experiments confirmed that the spectrum of *s*-like states deviates strongly from the 2D hydrogen-like series. Further experiments in magnetic fields reached up to 5s excited states [51,52,53,54,55], addressing also the complicated problem of the diamagnetic shifts of exciton lines resulting from three contributions (see Figure 1), as discussed theoretically in one of our other works [56]. The exciton series consists not only of bright *s*-series but also of two types of dark states—spin-forbidden and excited excitons states with symmetry different from ‘*s*’. The former one can be probed by PL dynamics [57,58], different emission configurations [59,60,61,62,63,64], the different temperature dependence of emissions [65], or by using a tilted magnetic field [60,66,67,68,69]. The latter ones are usually probed using two-photon spectroscopy [70,71]. Dark *p* states can also be studied in pump–probe experiments [72,73,74,75,76,77,78], where 1s excitons are generated and transition from 1s to 2p states, which can be measured by a probe terahertz beam [79]. Additionally, excitons with finite center-of-mass momentum, which require phonon-assisted excitation due to momentum conservation can also be probed, revealing the complicated landscape of so-called momentum-indirect excitons [80,81,82,83].

In addition to strongly bound excitons, TMDs exhibit another type of bound complexes in their photoresponse, namely trions [84,85,86,87]. In the simplified picture, this complex consists of three particles, i.e., two electrons + one hole (X${}^{-}$) or two holes + one electron (X${}^{+}$) for negatively and positively charged types, respectively. In GaAs quantum wells, the rule of thumb is that trion-binding energy is an order of magnitude smaller than that for exciton, and the same rule seems to work in TMDs. Because the binding energy of the exciton is on the order of hundreds of meV in TMDs, the trion-binding energy is given in tens of meV [88,89,90,91,92,93,94,95,96,97]. The existence and properties of such complexes depend heavily on the presence of excess carriers [13,36,91,98,99,100,101,102,103,104,105,106,107,108], and their stability has been shown up to room temperatures [88,97]. For higher doping regimes, a picture of an exciton interacting with the Fermi sea has been proposed [43,109]. Interestingly, just as in the exciton case, trions exhibit a fine structure [107,108,110,111,112,113,114,115,116], involving both dark/bright states and also an excited trion states [117]. Moreover, a strong electron–phonon interaction allows to couple trions and excitons by an up-conversion process [118,119], in which low-energy photon excitation (exciting trion) induces an optical emission from a higher energy state (exciton). It is worth noting that larger optical complexes have also been experimentally studied [104,110,120,121,122,123,124,125,126,127,128,129,130,131,132]. Their interpretation is, however, non-trivial due to the various possible couplings with other quasiparticles, e.g., related to excess charge carriers [86], collective excitations of electron gas [133,134], and polarons [43,135].

Atomically thin crystals offer a novel route to engineering material properties due to the possibility of assembling them into configurations not present in nature [136]. Starting from proof-of-concept double layers of graphene with hBN layers between them [137], it has been shown that graphene can act as an atomically sharp contact to TMDs [138]. Recently, one of the greatest improvements of the optical quality of semiconducting crystals was achieved by placing them in-between thin hBN crystals [16,139]. Different band gaps and band edge positions allowed for creating analogues of type I and type II heterostructures [140,141]. In such systems, we can distinguish two types of excitons: intralayer excitons, localised in one of the layers building the heterostructure, and interlayer excitons (sometimes called indirect), built by electrons and holes from distinct layers. Interlayer excitons have been observed [141,142,143,144,145,146,147,148,149,150,151,152,153,154,155,156,157,158], showing the electrical tunability of both PL and excitons energy [156,159,160,161]. Order-of-magnitude-longer lifetimes than intralayer excitons were observed due to the spatial separation of electron and hole [142,158,162]. Unlike interlayer excitons in GaAs heterostructures, for TMD heterostructures, we deal with interlayer excitons stable at room temperatures [160,163,164,165]. Larger complexes with carriers residing in different layers have already been proposed and some evidence of such behavior has been found [156,161,166,167,168,169]. Long interlayer exciton lifetimes naturally led to the proposal of exciton Bose condensates [164,165], with the first evidence in the MoSe${}_{2}$-WSe${}_{2}$ system [170]. It is worth noting that semiconductor heterostructures offer new means of control by the twist angle, an area that has been actively pursued in recent years [148,159,161,171,172,173,174,175,176,177,178,179,180,181,182,183,184,185,186,187,188,189,190,191,192].

### 1.3. Review of Theory of Correlated Optical Excitations

The successful description of bound excitonic states requires an analysis of two-particle correlations, described by the Bethe–Salpeter equation (BSE) [193,194,195,196,197,198,199,200]. Currently, several ab initio codes implement the DFT + GW + Bethe–Salpeter framework [201,202,203,204,205,206,207,208]. First-principles Green’s function theory of optical excitations can also be applied to materials with reduced dimensionality. Many studies of excitons in 2D semiconductors within DFT + GW + BSE have emerged [25,70,209,210,211,212,213,214,215,216,217,218,219,220,221,222,223,224,225]. Although common difficulties with the numerical aspects of excitons in TMDs [211,221] are known, those studies correctly identified that excitonic effects in TMDs should be large [25,209,210] due to the strong electron–hole interaction (weak screening) compared to bulk crystals. The inclusion of the spin-orbit coupling (SOC) confirmed the general double-peak structure of the optical response (A and B excitons), associated with the valence band split-off [226], as well as the fine structure related to the spin arrangement in the conduction band [227] and momentum-dark excitons [228]. The first ab initio calculations of the excited states of exciton series predicted large deviations from the non-hydrogenic series, the existence of 2p–2s splitting in the first shell [70], and the topological splitting of the 2p states [78]. Non-analytic (Dirac-like) dispersion of exciton-excited branches due to inter-valley coupling has been predicted [218,219]. The mixing of *A* and *B* excitons via the inter-valley exchange interaction has shown that understanding them in terms of “Ising” excitons may not always be satisfactory [225]. Interestingly, the DFT + GW + Bethe–Salpeter methodology has been recently extended to study charged exciton complexes [117,229,230,231].

Large differences between the binding energies of excitons on different substrates have been early established [215,217,220]. Interestingly, MoS${}_{2}$ put on the two different, most popular substrates, SiO${}_{2}$ and hBN, which have almost exactly the same energy in the optical gap. This is surprising because it is known that the binding energies of excitons on those substrates should be significantly different. Another confusing result is that the absolute peak position (G_0_W_0_ + BSE), almost identical to the experimental, is calculated for MoS${}_{2}$ in vacuum. The similarity of these values is coincidental and stems from the cancellation of self-energy and excitonic renormalizations, which are true even when the dielectric environment (vacuum, SiO${}_{2}$, hBN) is vastly different. Recent GW calculations of MoS${}_{2}$ on hBN [232] help to rationalize this, showing the actual position of the GW-normalized “free particle” band gap.

Similarly to the case of monolayers, many theoretical studies for TMD heterostructures emerged as well [223,226,233,234,235,236,237,238,239,240,241,242]. Bright interlayer excitons were predicted [236] with A and B exciton structures [223,226,233,236,237,238] analogous to the monolayer. It is worth noting that exciton binding energy is strongly correlated with the stacking and emergence of moiré patterns [233,237,238,239,240,241]. Furthermore, both the temperature dependence of the exciton fine structure [223,236,238] and the electrical control of exciton energy [234] were analyzed. Additionally, the spectrum of excited states was predicted [223] and the exciton lifetime was determined [241,243].

Due to many computational challenges inside DFT + GW + Bethe–Salpeter theory, simplified approaches to the excitonic problem have been studied, including various levels of approximations, i.e., tight-binding [79,80,82,83,244,245,246,247,248,249,250,251], *k·p* (massive Dirac fermion and beyond) [28,92,218,252,253,254,255,256,257,258,259], and effective mass approximations [47,53,139,260,261,262,263,264,265,266,267,268,269,270,271]. Those approximations routinely use DFT as a staring point for the calculation of the effective mass of carriers and then simplify somehow their interaction, e.g., assuming only Rytova–Keldysh screening [272,273] without form factors coming from the Bloch part of the electron and hole Bloch wave functions. These methods, being conceptually and numerically traceable, allowed for many fascinating advances in understanding exciton properties in TMDs. For the case of the heterostructure, *k·p* models were also developed, allowing studies of intra- and interlayer exciton properties [77,274,275,276,277,278,279,279]. Further simplification of the *k·p* methodology to the massive Dirac fermion model was used to analyze intra- and interlayer exciton properties [77] and the Bose–Einstein condensate of excitons [277,278,280].

Due to complicated numerical and conceptual challenges inside DFT + GW + BSE theory and the somehow approximate nature of the simplified methods mentioned above, one may turn to a tight-binding methodology, offering an optimal trade-off between numerical tractability and the ability to capture essential physical ingredients in the excitonic problem. Tight-binding studies [79,80,82,244,248,249,251,252,281] allowed, e.g., to track exciton fine-structure evolution dependence on electron doping, the exchange interaction, and magnetic and electric fields. Theoretical results on excitonic landscapes have been shown [83,247], together with results confirming the topological splitting of 2p states in the exciton spectrum due to the effect of the Berry’s curvature. In our own work [281], as further described in more detail, we studied the role of band nesting, screening, and wave function topology on exciton fine structure.

## 2. Electronic Structure of MX${}_{2}$ Semiconductors

### 2.1. Ab Initio Insight into Electronic Structure

**Figure 2:**
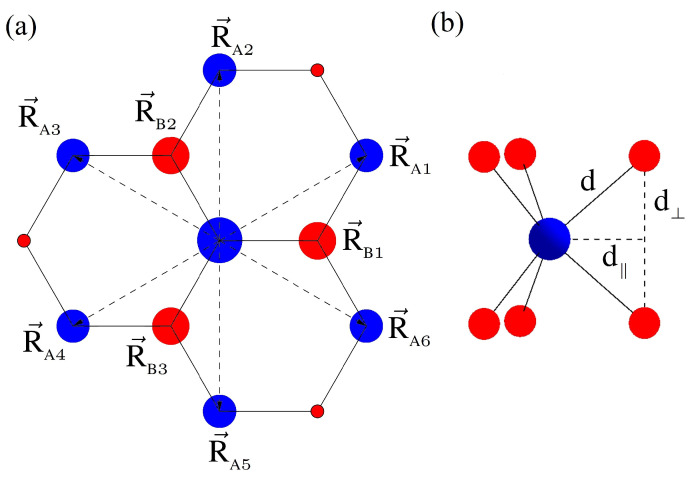
(**a**) Top view of the structure of MX${}_{2}$ in the 2H phase: metal atoms are denoted by blue dots, and chalcogen by red ones. (**b**) Side view of MX${}_{2}$, showing that the atoms are organized into three layers, central metal and two chalcogen, with structural constants parametrized by $d_{\|}$ and $d_{⊥}$.

In the following subsection, we review our understanding of TMD monolayers based on ab initio techniques that we used in several recent works [24,56,281,282]. Single-layer MX${}_{2}$ crystals in a 2H phase are arranged in a trigonal prismatic structure. The unit cell consists of three atoms, one metal and two chalcogens, as shown in Figure 2. From the top, the arrangement of the atoms reminds one of graphene. From the side, one can notice that the atoms are actually organized in three planes, one (central) metal plane and two chalcogen planes shifted by $\pm d_{⊥}$. The distance between the metal atom and the central position between the two chalcogens is denoted by $d_{\|}$. We define the primitive vectors of the real space lattice as ${\vec{a}}_{1}=d_{\|}\left(0,\sqrt{3}\right)$ and ${\vec{a}}_{2}=d_{\|}\left(3/2,-\sqrt{3}/2\right)$. The lattice constant $a_{0}=d_{M-M}$ can be written as $a_{0}=d_{\|}\sqrt{3}$ and $d_{⊥}=d_{X-X}/2$. Following the commonly practiced ab initio procedure, described in detail elsewhere [282], the lattice constants calculated using the PBE exchange-correlation functional for MoS${}_{2}$ are $d_{\|}=1.8393$$d_{⊥}=1.5622$ Å. Reciprocal lattice vectors $\vec{b}$ satisfy the relation $e^{i\vec{b}\cdot \vec{a}}=1$. This gives a 2D set of four equations ${\vec{b}}_{i}\cdot {\vec{a}}_{j}=2\pi \delta_{i,j}$ for i,j∈1,2. The solution yields ${\vec{b}}_{1}=2\pi /d_{\|}\left(1/3,1/\sqrt{3}\right)$ and ${\vec{b}}_{2}=2\pi /d_{\|}\left(2/3,0\right)$. We note that such choice of real and reciprocal space-primitive vectors gives the following position of the hexagonal Brillouin zone (BZ) corner at $K_{1}=\left(0,4\pi /\left(3\sqrt{3}d_{\|}\right)\right)$, which is called the *K* point, just as in graphene. All other *K* points are obtained by the successive application of the $C_{6}$ rotation. We note that in half of the distance between the *K* points and the Γ point there are so-called *Q* points (alternatively Σ [251] or Λ [268] points). Halfway between the two nearest *K* points lies the *M* points.

**Figure 3:**
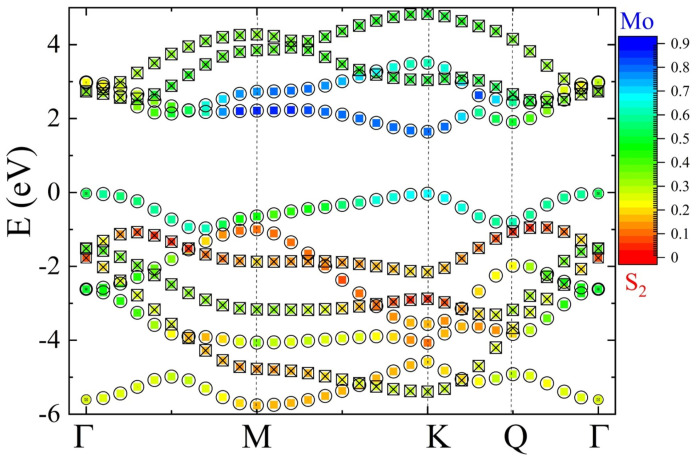
Color-mapped localization of a given k-resolved eigenenergy on Mo and S${}_{2}$ spheres and symmetry of eigenvalues across the Brillouin zone. Circles (crossed rectangles) denote symmetric (anti-symmetric) orbitals with respect to the metal plane.

Now, we describe the general features of the DFT band structure, focusing on a representative example of MoS${}_{2}$. In Figure 3, one can observe 11 bands around the Fermi level (set to 0). The direct band gap at the *K* point without SOC is equal to $\Delta_{\mathrm{GAP}}=1.67$ eV. We note that both the valence band (VB) and the conduction band (CB) have secondary extrema, at the Γ point in the VB and at the *Q* points in the CB. Next, we study the general properties of the DFT Kohn–Sham wave functions. First, we choose three spheres around one Mo and two S${}_{2}$ atoms, and calculate how much of the wave function is localized inside each sphere. We note that 11 bands around the Fermi level have wave functions that are in general localized on both Mo and S${}_{2}$ atoms; however, there is a clear trend that VB and bands above are mainly localized on Mo atoms, and VB-1 and lower bands on S${}_{2}$ atoms. DFT wave functions inside spheres can be next projected onto Slater orbitals. We first confirm that the largest overlap is achieved, in case of MoS${}_{2}$, with localized Slater, single-zeta-basis-consistent [283,284] with orbitals with a principal quantum number n=4 on Mo and n=3 on S. For other compounds, e.g., WSe${}_{2}$, there is a clearer and larger numerical overlap with orbitals with quantum numbers n=5 for W and n=4 for Se, confirming that such projection is a reliable tool in studying symmetry and the orbital composition properties of DFT wave functions. In the next step, summarized in Figure 3, for each wave vector $\vec{k}$ and energy *E*, the wave functions are projected onto symmetric and anti-symmetric orbitals with respect to the metal plane. One can observe that VB and CB are symmetric across most of the Brillouin zone, with the exception of parts close to the Γ point in the CB. This ordering of bands and their symmetry is general for all MX${}_{2}$-family compounds. In the next step, we study the orbital-resolved decomposition of wave functions. The results confirm that orbital composition close to the *K* point in the VB bands is a combination of $4d_{+2}$ and $3p_{+1}$ orbitals. Contrary to VB, in the CB, the $4d_{0}$ state coupled to the $3p_{-1}$ state dominates. The higher symmetric conduction band (CB + 1) ($4d_{-2}$) is coupled to the $3p_{0}$ state. Coupling between anti-symmetric *d* and *p* states is also obtained, in which the $4d_{-1}$ state is coupled to anti-symmetric $3p_{+1}$ and $4d_{+1}$ to both anti-symmetric $3p_{-1}$ and $3p_{0}$ states.

Due to the heavy nature of the atoms in the MX${}_{2}$ family, one can expect that a relativistic spin-orbit interaction may influence the band structure. Here, we discuss briefly the results of the PBE + SOC calculation for MoS${}_{2}$. The main effect of SOC is visible in the VB at *K* point, where bands are spin-split by $\Delta_{VB}^{SOC}=148$ meV. It is worth mentioning that this is the smallest splitting in the VB at the *K* point in MX${}_{2}$. Significantly smaller splitting is observed in the CB at *K* point in MoS${}_{2}$ ($\Delta_{CB}^{SOC}=3$ meV). This less-pronounced order-of-magnitude effect comes from the fact that the majority of (80%) *d*-like orbitals in the CB have the quantum number $m_{d}=0$, therefore splitting must come from the admixture of *p*-orbitals (10%) from chalcogen atoms. What is also important is that there is a significant spin splitting in the second minimum of the CB at *Q* point, which is larger than at *K* point, pushing the relative distance between the CB minimum at *K* at *Q* point ($\Delta_{K-Q}^{SOC}$) close to small values, making the CB almost degenerate, e.g., in WSe${}_{2}$. Let us also note that by symmetry there is no spin splitting along the Γ−M line in the BZ.

### 2.2. Minimal Tight-Binding Hamiltonian

In the following subsection, we review our construction of a tight-binding model for TMDs monolayers [56,282]. Building on our DFT analysis presented in the previous subsection, we conclude that there are several common features in the band structures of analyzed MX${}_{2}$ semiconductors. They have a direct band-gap at *K* points, opposite to their N≥2-layer form, which are indirect-gap semiconductors [24]. These materials also have secondary minima in the conduction band, localized close to the *Q* points, and secondary maximum in the valence band at the Γ point. Analyzing orbital compositions, we conclude that CB and VB can be described by orbitals, even with respect to the metal plane. This result is consistent with several other works [285,286,287,288,289,290,291,292,293,294,295]; therefore, we can start building a tight-binding model of those materials from the orbitals contributing most to the band structure around the Fermi level.

Our conclusion is that the minimal tight-binding model has to include orbitals that are even with respect to the inversion symmetry about the *z* plane of the metal atoms (z→−z). Motivated by ab initio results, at least m=0,±2*d*—orbitals from metals—and m=0,±1 symmetric *p*-orbitals from the top and bottom sulfur atoms must be taken into account. We note only that for the *d* orbitals of metals the situation is clear, namely orbitals with a given L=2 and m=−2,0,+2 are centered around M atoms. However, the orbital construction for two chalcogens must be performed with care. Because we define the so-called dimer orbitals, which are centered around the same plane as metal atoms, we begin with upper (U, ${\vec{R}}^{U}=\left(0,0,+d_{⊥}\right)$ with respect to the dimer center) and lower (L, ${\vec{R}}^{L}=(0,0,-d_{⊥}$)) *p*-orbitals with quantum numbers L=1, m=±1 and define dimer orbitals φ as a proper combination of those two, as described in detailed elsewhere [56,282].

In the next step, we construct the standard linear combination of the atomic orbitals for all of the orbitals discussed above. First, we note that due to our dimer orbital construction, it makes sense now to talk about the sublattices A (metal positions, $\tau_{1}=\left(0,0,0\right)$ inside the unit cell) and B (chalcogen dimer centers, $\tau_{2}=\left(d_{\|},0,0\right)$) on the real-space hexagonal lattice. Because we aim to understand the fundamental properties of those materials in terms of graphene physics, we focus on the sublattice A–sublattice B interaction. First, let us try to understand the physics of why the MX${}_{2}$ materials considered are semiconductors instead of semimetals, such as graphene. To achieve that, let us analyze the tunneling matrix element between the central A atom with the potential $V_{A}(\vec{r})$ and the three nearest neighbor B atoms at positions ${\vec{R}}_{B_{1}},{\vec{R}}_{B_{2}},{\vec{R}}_{B_{3}}$. One can notice, that exactly at the *K* point, this formula gives

(1) $$\langle \Psi_{A,m_{{}_{d}}}^{\vec{k}=K}|\hat{H}|\Psi_{B,m_{{}_{p}}}^{\vec{k}=K}\rangle =\left(1+e^{i\left(1-m_{d}+m_{p}\right)2\pi /3}+e^{i\left(1-m_{d}+m_{p}\right)4\pi /3}\right)V_{pd},$$

where $V_{pd}$ are standard Slater–Koster integrals. For such combinations of $m_{d}$ and $m_{p}$ quantum numbers, that $1+m_{p}-m_{d}=0,\pm 3$ tunneling matrix element is non-zero $\langle \Psi_{A,m_{{}_{d}}}^{\vec{k}=K}|\hat{H}|\Psi_{B,m_{{}_{p}}}^{\vec{k}=K}\rangle \neq 0$. This is different from the same tunneling matrix element in graphene, in which only L=1, $m_{p}=0$$p_{z}$ orbitals play a role and give $\langle \Psi_{A,m_{{}_{p}}}^{\vec{k}=K}|\hat{H}|\Psi_{B,m_{{}_{p}}}^{\vec{k}=K}\rangle =0$. Non-vanishing tunneling will, therefore, open a gap at the *K* point, resulting in a gapped, massive Dirac fermion dispersion instead of the Dirac point at *K*. Non-zero interaction between different $m_{d}$ and $m_{p}$ orbitals at *K* point leads to the following pairs of orbitals that are coupled: $\left[m_{d}=-2,m_{p}=0\right]$, $\left[m_{d}=0,m_{p}=-1\right]$, and $\left[m_{d}=2,m_{p}=1\right]$. Similar analysis at −K point leads to the selection rule $-1+m_{p}-m_{d}=0,\pm 3$. The resulting couplings are then $\left[m_{d}=-2,m_{p}=-1\right]$, $\left[m_{d}=0,m_{p}=1\right]$, and $\left[m_{d}=2,m_{p}=0\right]$. Finally, at Γ point, we obtain a different scheme of couplings: $\left[m_{d}=0,m_{p}=0\right]$, $\left[m_{d}=2,m_{p}=-1\right]$, and $\left[m_{d}=-2,m_{p}=1\right]$. We note that all those couplings explain the couplings detected in DFT.

In next step, we move to a discussion of the tight-binding Hamiltonian. The standard procedure outlined by Slater and Koster (SK) [296] is used. All details of this straightforward derivation are discussed elsewhere [56,282]. We note only that because in our basis we are using complex orbitals, SK formulas cannot be used directly and we had to implement them into a more complicated fashion, which is hidden in our notation behind functions V,W, depending on the SK parameters. The next-nearest neighbor Hamiltonian can be written in block form as:

(2) $$H\left(\vec{k}\right)=\left[\begin{array}{cc} H_{M-M} & H_{M-X_{2}}\\ H_{M-X_{2}}^{†} & H_{X_{2}-X_{2}} \end{array}\right]$$

where the matrix describing the metal–metal next-nearest neighbor interactions is given by

(3) $$H_{M-M}=\left[\begin{array}{ccc} {}_{+W_{1}g_{0}\left(\vec{k}\right)}^{E_{m_{{}_{d}}=-2}} & W_{3}g_{2}\left(\vec{k}\right) & W_{4}g_{4}\left(\vec{k}\right)\\ \; & {}_{+W_{2}g_{0}\left(\vec{k}\right)}^{E_{m_{{}_{d}}=0}} & W_{3}g_{2}\left(\vec{k}\right)\\ \; & \; & {}_{+W_{1}g_{0}\left(\vec{k}\right)}^{E_{m_{{}_{d}}=2}} \end{array}\right],$$

and the corresponding matrix describing the X${}_{2}$–X${}_{2}$ dimer interactions is given by

(4) $$H_{X_{2}-X_{2}}=\left[\begin{array}{ccc} {}_{+W_{5}g_{0}\left(\vec{k}\right)}^{E_{m_{{}_{p}}=-1}} & 0 & -W_{7}g_{2}\left(\vec{k}\right)\\ \; & {}_{+W_{6}g_{0}\left(\vec{k}\right)}^{E_{m_{{}_{p}}=0}} & 0\\ \; & \; & {}_{+W_{5}g_{0}\left(\vec{k}\right)}^{E_{m_{{}_{p}}=1}} \end{array}\right],$$

Finally, metal dimer tunneling is described by

(5) $$H_{M-X_{2}}=\left[\begin{array}{ccc} V_{1}f_{-1}\left(\vec{k}\right) & -V_{2}f_{0}\left(\vec{k}\right) & V_{3}f_{1}\left(\vec{k}\right)\\ -V_{4}f_{0}\left(\vec{k}\right) & -V_{5}f_{1}\left(\vec{k}\right) & V_{4}f_{-1}\left(\vec{k}\right)\\ -V_{3}f_{1}\left(\vec{k}\right) & -V_{2}f_{-1}\left(\vec{k}\right) & -V_{1}f_{0}\left(\vec{k}\right) \end{array}\right],$$

The functions *f* and *g*, depending on the wave vector $\vec{k}$, combine the proper sum of the plane wave functions to the nearest and next-nearest neighbors. They are listed in Ref. [56]. As discussed in one of our works [56], we find that the next-nearest neighbor Hamiltonian is a minimal model that correctly describes the band gap across the whole BZ and is able to quantitatively reproduce the orbital compositions of the VB and CB. We note that the last column (except the diagonal element) in the Hamiltonian has a sign opposite to the one used in Ref. [56] due to two different conventions of complex spherical harmonics that could be used (Condon–Shortley phase). Here, complex and real orbitals are related by $\varphi_{m_{p=\pm 1}}=\pm 1/\sqrt{2}\left(\varphi_{p_{x}}\pm i\varphi_{p_{x}}\right)$.

After the derivation of our tight-biding Hamiltonian, we turn to the problem of fitting the SK parameters. Our goal is to obtain dispersion for even bands from TB as close as possible to even bands obtained and detected using the ab initio method, as described above. We note that in principle it is possible to calculate SK parameters directly from a first-principles calculation [297]; however, the resulting electronic dispersions are not satisfactory, neither for graphene [298], nor for MoS${}_{2}$ [289], especially for unoccupied electronic states (CB and higher bands). Those parameters could be in principle treated as a starting point for our analysis; however, we take another route. The general problem with SK parameters is that even if we fix the structural parameters $d_{⊥}$ and $d_{\|}$, we still end up with a 10-dimensional, highly non-linear optimization problem, depending on the energies $E_{m_{d}=0}=E_{m_{d}=\pm 2}$, $E_{m_{p}=0}$, $E_{m_{p}=\pm 1}$, $V_{dp\sigma }$, $V_{dp\pi }$, $V_{dd\sigma }$, $V_{dd\pi }$, $V_{dd\delta }$, $V_{pp\sigma }$, and $V_{pp\pi }$. We have tested various schemes to choose those parameters to fit the dispersion of such a model to the DFT [282]. At the end of the day, we used the one closest to the Monte Carlo philosophy of random probing of multidimensional parameter space.

**Figure 4:**
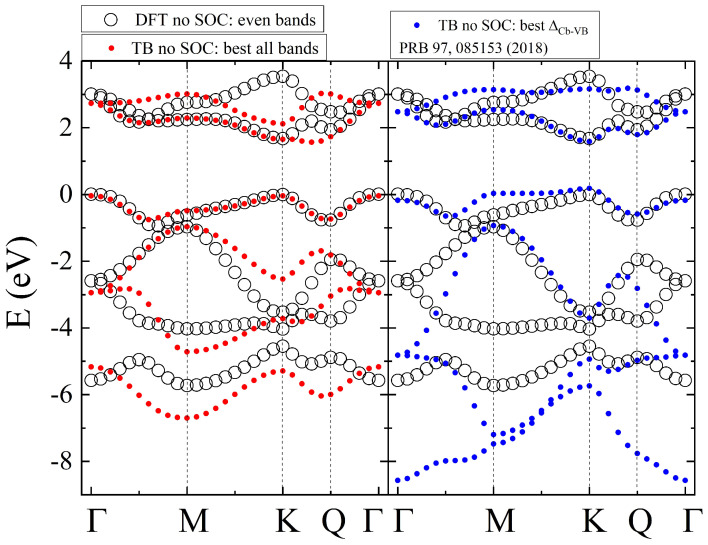
**Left**: TB dispersion obtained after optimizing the SK parameters to reproduce all even DFT bands. **Right**: TB dispersion optimized to reproduce the transition energy between the VB and CB. We note that the former reproduces the VB very well, while the latter one reproduces the CB very well, especially on the K−Γ line.

The results of two fitting procedures are presented in Figure 4. We note that the fit presented in the left of Figure 4 was found by assuming equal weights for all *k* points and all even bands; therefore, it is called “best all bands”. Additionally to reproducing the overall energies of even bands well, this parametrization reproduces VB very well. To obtain the best transition energy (right panel of Figure 4), a much more complicated procedure was used, first, increasing the weights for VB and CB on the entire Γ−M−K−Γ line, then, in subsequent sweeps, the weights around the *K* point in the VB and CB and the *Q* point in the CB were further increased. Thanks to those weighting procedures, both the VB and CB along the K−Γ line are reproduced very well.

To include spin-orbit coupling in our tight-binding model, we analyze the matrix elements of the $\vec{L}\cdot \vec{S}$ operator. It turns out that only non-zero spin-orbit operator matrix elements are diagonal in our basis and read (for spin-up)

(6) $${\hat{H}}_{SOC}=\mathrm{diag}\left(-\lambda_{M},0,\lambda_{M},-\frac{1}{2}\lambda_{X_{2}},0,\frac{1}{2}\lambda_{X_{2}}\right).$$

The full Hamiltonian with SOC can be written therefore as:

(7) $$H_{\mathrm{TB}+\mathrm{SOC}}\left(\vec{k}\right)=H\left(\vec{k}\right)⊗\left[\begin{array}{cc} 1 & 0\\ 0 & 1 \end{array}\right]+H_{SOC}⊗\left[\begin{array}{cc} 1 & 0\\ 0 & -1 \end{array}\right].$$

The parameters $\lambda_{Mo}$ and $\lambda_{S_{2}}$ have to be chosen in order to reproduce the spin splitting of the bands. This choice in general changes the dispersion of the bands due to the modification of the diagonal parts of the Hamiltonian and, in principle, requires additional fitting. We checked that setting $\lambda_{Mo}=0.148/2$ eV and $\lambda_{S_{2}}=0.03/2$ reproduces the correct splittings in the VB (0.148 eV) and CB (0.003 eV). We note that the order-of-magnitude-higher value of $\lambda_{S_{2}}$ stems from a different contribution of $m_{p}\neq 0$ orbitals to CB (order of 20%).

**Figure 5:**
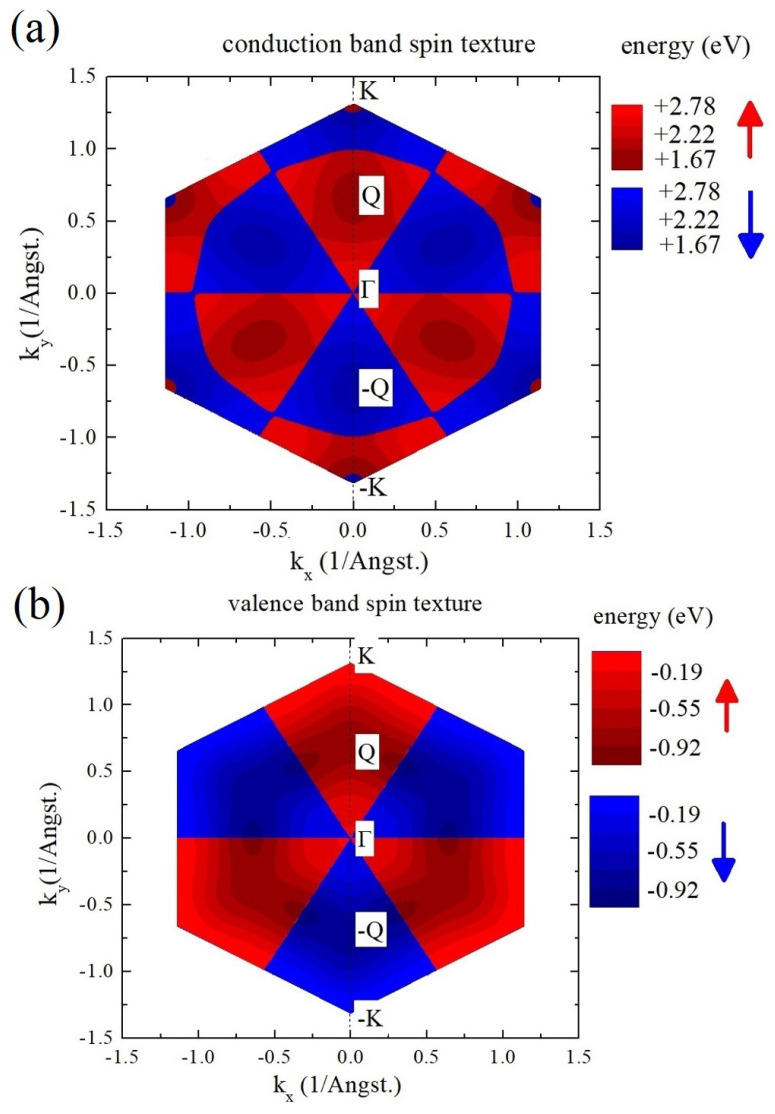
(**a**) Spin texture of the CB, showing the spin orientation of the lowest CB spin-split band. (**b**) Corresponding VB spin texture.

To better understand how SOC affects the band structure across the BZ, let us analyze spin-split bands along the (+K)−Γ−(−K) direction. Our choice of parameters reproduces spin splitting in the VB and CB at *K* points well and our model catches spin inversion between the +K and −K points. Interestingly, the general feature of the whole MX${}_{2}$ family is the spin inversion of bands in the CB close to the *K* point, taking place between *K* and *Q* points. This feature is better visible when the lowest spin-split band is shown throughout the BZ, as shown in Figure 5a,b. For example, in the +K point, the bottom of the CB has a spin orientation the same as the top of the VB. However, the region of the BZ where this property holds is very small (red region around +K in Figure 5b), and quickly, the other spin becomes lower in energy. This situation changes again approximately half-way between the *K* and *Q* points, around which the spin is again oriented in the same way as at the +K point in the VB. Interestingly, in both Mo and W-based TMDs, the spin at *Q* point is always oriented the same way as in the VB at *K* point, irrespective of the spin ordering change in W-based materials. The same spin orientation between the VB at *K* and *Q* at the CB means that all momentum-indirect excitons with momentum |Q−K| will have a spin-“bright” configuration and, when activated, e.g., by phonons, they should be optically detectable, in contrast to the spin-forbidden lowest excitons that have a small momentum around *K* points in tungsten-based TMDs.

In the next step, we discuss the low-energy Hamiltonian around *K* point. We begin by noticing that at *K* point, the top of the VB is built almost solely from the $m_{d}=+2$ orbital, while the bottom of the CB is built from a combination of $m_{d}=0$ and $m_{p}=-1$ orbitals. However, assuming the low-energy basis as $m_{d}=0$ and $m_{d}=+2$, and expanding $g_{0}$ and $g_{2}$ functions around *K* point ($\vec{k}=\vec{K}+\vec{q}$), one can immediately end up with a massive Dirac fermion (mDF) Hamiltonian [299]:

(8) $$H_{mDF}=at\left(\begin{array}{cc} 0 & q_{x}-iq_{y}\\ q_{x}+iq_{y} & 0 \end{array}\right)+\frac{\Delta }{2}\left(\begin{array}{cc} 1 & 0\\ 0 & -1 \end{array}\right)$$

The best parametrization for dispersion including up to 1/4 distance between the *K* and *Q* point we found is Δ=1.6848 eV, a=3.193 Å, t=1.4677 eV. The simple mDF model defined by Equation (8) gives very similar results of dispersion compared to models with further corrections, such as trigonal warping, and when applied to excitonic calculations there is no qualitative and very little quantitative difference. We note that mDF can be even further reduced to the parabolic (effective mass) model. This can be performed by noting that eigenergies of mDF are:

(9) $$E=\pm \sqrt{\frac{\Delta^{2}}{4}+a^{2}t^{2}q^{2}}=\pm \frac{\Delta }{2}\sqrt{1+\underset{\varepsilon }{\underset{︸}{\frac{4a^{2}t^{2}}{\Delta^{2}}q^{2}}}}\underset{\varepsilon <<1}{\underset{︸}{\approx }}\pm \;\frac{\Delta }{2}\left(1+\frac{1}{2}\varepsilon -\frac{1}{8}\varepsilon^{2}+\ldots \right)$$

Keeping only the first order of ε, we end up with

(10) $$E=\pm \left(\frac{\Delta }{2}+\frac{\hbar^{2}q^{2}}{2m^{*}}\right).$$

The effective electron mass is given as $m^{*}=\Delta \hbar^{2}/\left(2a^{2}t^{2}\right)$. We note that the choice of parameters $\Delta ,t,m^{*}$ depends heavily on how large a portion of the BZ we want to fit as closely as possible. One can also note that the top of the VB and the bottom of the CB are described better if different effective masses of electrons ($m_{e}$) and holes ($m_{h}$) are taken. For MoS${}_{2}$, when using the parabolic model, we take $m_{e}=0.54\;m_{0}$ and $m_{h}=0.44\;m_{0}$.

**Figure 6:**
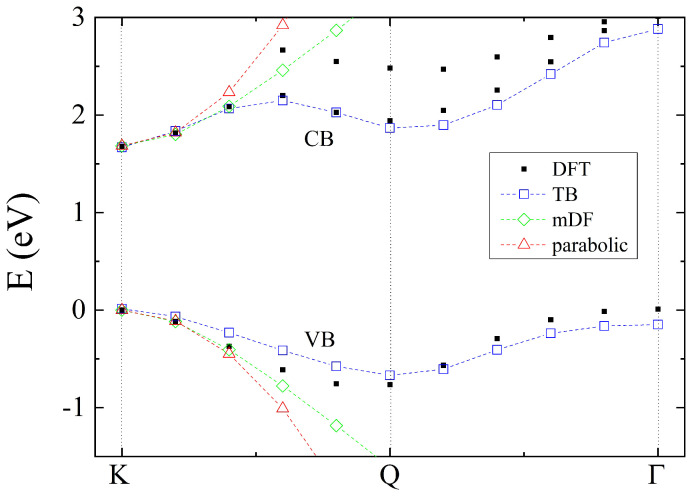
Comparison between dispersion models along the K−Γ line. DFT dispersion is denoted by black circles, TB by blue rectangles, massive Dirac fermion by green diamonds, and parabolic (effective mass) model by red triangles. The corresponding connecting lines are shown as a guide to the eye.

As a summary, in Figure 6, we present the ab initio result and different low-energy models of the VB and CB: the tight-binding, massive Dirac fermion, and parabolic models. One can observe that all of them describe well the neighborhood of *K* point; however, by only using the TB model it is possible to obtain a second minimum at the *Q* point in the CB and a correct second maximum of the VB at the Γ point. Approximately 10% of the K−Γ line from the *K* point is properly described by both mDF and parabolic models, with the mDF model being better for the CB description. We note also that both mDF and parabolic models can be extended to include spin; however, in both cases it is necessary to find a different parametrization for two spin species (e.g., two effective electron and hole masses and two gap parameters Δ for the parabolic model). Such spin-dependent low-energy model parameters for MX${}_{2}$ semiconductors are also available in the literature [300].

## 3. Tight-Binding Theory of Optical Excitations

### 3.1. Bethe–Salpeter Equation

The simplest picture of light absorption by semiconducting material can be understood in terms of the transitions of carriers from the valence to the conduction band due to photon excitation with energy $E_{exc.}$, changing the angular momentum by ±1 when circularly polarized light is used. In MX${}_{2}$ semiconductors, the situation becomes more complicated, because dipole transitions between d orbitals ($m_{d}=\pm 2$ in the VB and $m_{d}=0$ in the CB) and negligible *p*–*d* transitions cannot explain that circularly polarized light excites carriers within one valley. The solution to this problem comes from a realization that the velocity matrix elements inside the transition matrix elements in general have two contributions [301]: dipole transitions between localized orbitals and terms related to electron hopping between lattice sites. This hopping contribution sets the phase of the velocity matrix element between the VB and CB Bloch wave functions and defines the optical selection rules in the gapped “chiral” fermion systems [302,303].

**Figure 7:**
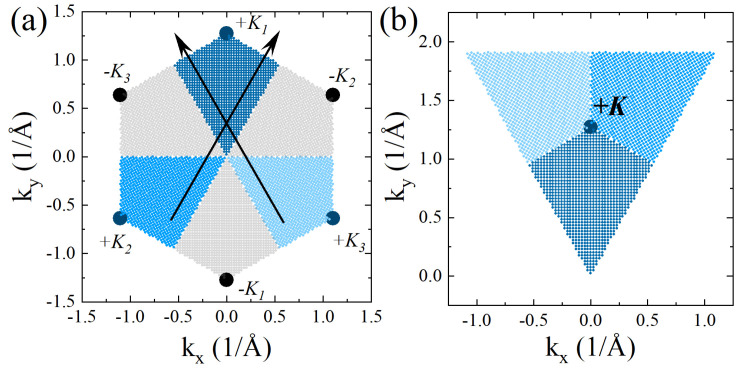
(**a**) Choice of +K valley on the whole BZ. (**b**) Construction of the valley around a single *K* point.

Because it is possible to selectively excite carriers from the band edge of one valley (e.g., +K), in theoretical investigations it is useful to think about two non-equivalent parts of the hexagonal BZ. A unique association of *k* points that belong to one or the other valley has to be performed, as shown in Figure 7. Starting from cutting “wedges” around three equivalent (related to each other by reciprocal lattice vector G translations) *K* points, as shown in Figure 7a, one can move respective wedges to one neighborhood of the *K* point, creating a triangle around it, as in Figure 7b. We note that a similarly constructed triangle for the −K valley has to be rotated by $C_{3}$ symmetry, and both triangles for +K and −K valleys placed next to each other create a rhomboidal BZ equivalent to a full hexagonal BZ.

Exciton properties can be calculated from the configuration-interaction approach to interacting electrons [304,305], particularly useful in studies of nanostructures, e.g., quantum dots [306,307,308,309,310,311,312,313,314,315,316,317,318,319,320,321,322] or electron gas in quantum wells in strong magnetic fields [323,324]. This approach has only recently been utilized to study excitons in reciprocal space [230,281].

**Figure 8:**
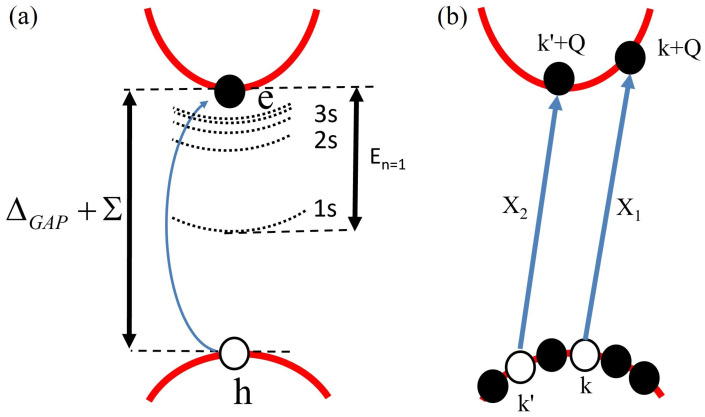
(**a**) The single electron–single hole picture (exciton in effective mass approximation) in which interaction creates a spectrum of bound states. (**b**) Exact picture where the “hole” is created by exciting the electron from the filled ground state in the VB. The exciton is then constructed as a coherent superposition of all possible excitations for a given center-of-mass momentum ${\vec{Q}}_{CM}$ interacting via the Coulomb interaction.

Now, we give a brief summary of steps performed for the derivation of the Bethe–Salpeter equation for the bound electron–hole problem, shown schematically in Figure 8a. We note that the complete derivation is presented in detail in Refs. [281,282]. We assume that we have only one valence and one conduction band. Next, to construct the ground state, we fill all states in the valence band, as shown in Figure 8b. Then, the exciton state is formed as a linear combination of excitations with coefficients $A_{n}^{Q_{CM}}$ being complex electron–hole amplitudes. Exciton states can be calculated using the standard eigenvalue problem ${\hat{H}}_{X}{|X\rangle }_{n}=E_{n}{|X\rangle }_{n}$, where ${\hat{H}}_{X}$ is an interacting excitonic Hamiltonian. After calculating the matrix elements of this Hamiltonian using the Wick theorem, neglecting ground state energy correction due to interactions and incorporating electron and hole self-energies into dispersion energies $\varepsilon_{c,k}$ and $\varepsilon_{v,k}$, we obtain the Bethe–Salpeter equation for the exciton (for center-of-mass momentum $Q_{CM}=0$)

(11) $$\left(\varepsilon_{c,k}-\varepsilon_{v,k}\right)A_{n}\left(\vec{k}\right)+\sum_{k^{\prime }}^{\mathrm{BZ}}\left[\begin{array}{c} -\langle v,\vec{k^{\prime }}\left|c,\vec{k}\left|V\right|c,\vec{k^{\prime }}\right|v,\vec{k}\rangle \\ +\langle v,\vec{k^{\prime }}\left|c,\vec{k}\left|V\right|v,\vec{k}\right|c,\vec{k^{\prime }}\rangle \end{array}\right]A_{n}\left(\vec{k^{\prime }}\right)=E_{n}A_{n}\left(\vec{k}\right)$$

In this equation, the summation over the $\vec{k^{\prime }}$ states is understood as over all vectors in the first BZ, the same as the number of unit cells in the crystal. Electron–hole interaction matrix elements are generally defined as:

(12) $$\begin{array}{cc} \; & \langle n_{1},{\vec{k}}_{1}\left|n_{2},{\vec{k}}_{2}\left|V\right|n_{3},{\vec{k}}_{3}\right|n_{4},{\vec{k}}_{4}\rangle =\\ \; & =\int_{R^{3}}d^{3}r\int_{R^{3}}d^{3}r^{\prime }\psi_{n_{1}}^{*}\left({\vec{k}}_{1},\vec{r}\right)\psi_{n_{2}}^{*}\left({\vec{k}}_{2},{\vec{r}}^{\prime }\right)V\left(\left|\vec{r}-{\vec{r}}^{\prime }\right|\right)\psi_{n_{3}}\left({\vec{k}}_{3},{\vec{r}}^{\prime }\right)\psi_{n_{4}}\left({\vec{k}}_{4},\vec{r}\right) \end{array}$$

We note that in our Equation (11), we expand the exciton wave function only in electron–hole pair excitations from the VB to CB and not reverse (just as Equation (16) in Ref. [200]). Such an expansion is called the Tamm–Dancoff approximation. If the interaction matrix elements are not screened, Equation (11) is called time-dependent Hartree–Fock. In our work, we always used screened versions of matrix elements (see discussion below); therefore, in our case, formally, we should be using the name simplified Bethe–Salpeter (simplified in the sense of using an approximate, not self-consistent, screening). In our approach, based on the configuration interaction picture, it is natural to use the same screening for both direct and exchange electron–hole terms, producing results that are consistent with other similar works [230].

**Figure 9:**
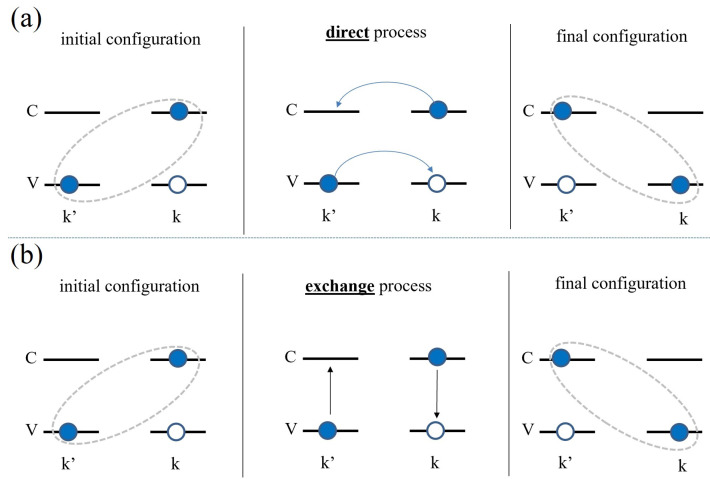
Graphical representation of two types of interaction between an electron and a hole: (**a**) direct process (**b**) exchange process.

We note that in general the issue of the screening of the exchange electron–hole interaction is a long-standing problem, with several works discussing its various aspects (see, e.g., references in Ref. [230]). It is understood from a formal point of view that the iterative solution for the two-particle Green’s function $G=G_{0}+G_{0}\cdot K\cdot G$ ($G_{0}$ is the non-interacting Green’s function describing the independent propagation of electron and hole, *K* is interaction kernel) does not allow the screening of the exchange electron–hole part of K, as discussed in Ref. [325]. However, there are reasons to screen it just as one direct stemming, e.g., from the finite Hilbert space of the problem when the Bethe–Salpeter equation is solved in practice. The screening of exchange in DFT + GW + Bethe–Salpeter has recently been discussed in the context of excitons in MoS${}_{2}$ monolayers in Refs. [326,327]. In an alternative approach to this problem, in paper [328], some of us expanded the systematically excited states of a graphene quantum dot in multi-electron–hole pair excitations. Figure 7 shows the evolution of ground and excited states as a function of the number of excited pairs. Figure 9 shows the singlet–triplet splitting. The main result is obtained by including an extra two pairs which do the screening, i.e., produce an effective interaction for singlet and triplet excitations.

### 3.2. Coulomb Matrix Elements

Let us identify the type of matrix elements within the summation over $\vec{k^{\prime }}$ in Equation (11). Note that they are written in electron-only language. As shown in Figure 9, the first element (with the minus sign) describes the process in which electrons in conduction bands in state $\vec{k}$ and electrons in valence bands in state $\vec{k^{\prime }}$ scatter via the Coulomb interaction to electrons in state $\vec{k^{\prime }}$ in conduction bands and electrons in state $\vec{k}$ in valence bands. This description is equivalent to the electron–hole pair scattering from state $\vec{k}$ to $\vec{k^{\prime }}$. The second process in Equation (11) describes electrons starting as previously in the $\vec{k}$ state in the conduction band and the $\vec{k^{\prime }}$ state in the valence band and scattering to the same $\vec{k}$ and $\vec{k^{\prime }}$, but changing band indices to valence and conduction, respectively. We identify the first process as an attractive direct electron–hole interaction, and the second process as a repulsive-exchange electron–hole interaction.

Next, we analyze direct electron–hole Coulomb matrix elements. Substituting the Bloch form of the wave functions and utilizing the fact that functions of coordinates in plane can be analyzed in reciprocal space (interaction and co-densities), we use their Fourier components as a general strategy to re-group six-dimensional integrals. The final expression for the direct matrix element (with coefficient $S/{\left(2\pi \right)}^{2}$ resulting from sum to integral transition) is

(13) $$V^{D}\left(\vec{k},\vec{k^{\prime }}\right)=\frac{S}{{\left(2\pi \right)}^{2}}\langle v,\vec{k^{\prime }}\left|c,\vec{k}\left|V\right|c,\vec{k^{\prime }}\right|v,\vec{k}\rangle =\gamma \sum_{\vec{G}}\frac{F^{D}\left(\vec{k},\vec{k^{\prime }},\vec{G}\right)}{\left|\vec{k^{\prime }}-\vec{k}-\vec{G}\right|},$$

where $\gamma =e^{2}/\left(8\pi^{2}\varepsilon_{0}\right)$ and interaction form factor $F^{D}$ is given by:

(14) $$F^{D}\left(\vec{k},\vec{k^{\prime }},\vec{G}\right)=\int dz\int dz^{\prime }\rho_{vv}^{\vec{k^{\prime }}\vec{k}}\left(\vec{G},z\right)\rho_{cc}^{\vec{k}\vec{k^{\prime }}}\left(-\vec{G},z^{\prime }\right)e^{-\left|z-z^{\prime }\right|\cdot \left|\vec{k^{\prime }}-\vec{k}-\vec{G}\right|}.$$

Pair densities can be evaluated numerically for every coordinate *z* by using the explicit form of the Bloch wave functions, constructed using localized Slater orbitals φ, as described before, as

(15) $$\begin{array}{cc} \rho_{vv}^{\vec{k^{\prime }}\vec{k}}\left(\vec{G},z\right)= & \frac{1}{N_{\mathrm{UC}}}\sum_{\alpha ,\beta =1}^{2}\sum_{\mu ,\nu =1}^{3}{\left[v_{\alpha \mu }^{\mathrm{VB}}\left(\vec{k^{\prime }}\right)\right]}^{*}v_{\beta \nu }^{\mathrm{VB}}\left(\vec{k}\right)\times \ldots \\ & \sum_{i,j=1}^{N_{\mathrm{UC}}}\exp\left[-i\vec{k^{\prime }}\cdot \left({\vec{U}}_{i}+{\vec{\tau }}_{\alpha }\right)+i\vec{k}\cdot \left({\vec{U}}_{j}+{\vec{\tau }}_{\beta }\right)\right]\times \ldots \\ & {\;\int \int \;}_{R^{2}}d^{2}r\left\{e^{-i\left(\vec{G}-\vec{k^{\prime }}+\vec{k}\right)\cdot \vec{r}}\varphi_{\alpha \mu }{\left(\vec{r}-{\vec{U}}_{i}-{\vec{\tau }}_{\alpha },z\right)}^{*}\varphi_{\beta \nu }\left(\vec{r}-{\vec{U}}_{j}-{\vec{\tau }}_{\beta },z\right)\right\}. \end{array}$$

We note that further discussions of the details of the properties of the direct Coulomb interaction, e.g., the behavior of pair densities, convergence issues, matrix elements symmetries, complex phase properties, and descriptions of their inter-valley behavior, are described elsewhere [281,282].

Analogously, the exchange Coulomb matrix elements are expressed by

(16) $$V^{X}\left(\vec{k},\vec{k^{\prime }}\right)=\frac{S}{{\left(2\pi \right)}^{2}}\langle v,\vec{k^{\prime }}\left|c,\vec{k}\left|V\right|v,\vec{k}\right|c,\vec{k^{\prime }}\rangle =\gamma \sum_{\vec{G}\neq 0}\frac{F^{X}\left(\vec{k},\vec{k^{\prime }},\vec{G}\right)}{\left|\vec{G}\right|},$$

with form factors

(17) $$F^{X}\left(\vec{k},\vec{k^{\prime }},\vec{G}\right)=\int dz\int dz^{\prime }\rho_{vc}^{\vec{k^{\prime }}\vec{k^{\prime }}}\left(\vec{G},z\right)\rho_{cv}^{\vec{k}\vec{k}}\left(-\vec{G},z^{\prime }\right)e^{-\left|z-z^{\prime }\right|\cdot \left|\vec{G}\right|}.$$

There are several differences between direct and exchange electron–hole interactions. As can be seen from Equation (11), direct interaction comes with a negative sing (electron–hole attraction) and an exchange one comes with a positive sign (electron–hole repulsion). Contrary to direct matrix elements, there is no $1/|k-k^{\prime }|$ overall dependence of magnitude of those elements; therefore, potentially exchange interaction can be large whenever direct matrix elements are small due to the large $|k-k^{\prime }|$ distance. We note also that interaction form factors $F^{D}$ consist of a pair of densities diagonal in band indices ($\rho_{vv/cc}$) and off-diagonal with wave vector indices ($\rho^{kk^{\prime }}$), while for exchange-interaction form factors, the situation is opposite, i.e., there is off-diagonal dependence on band indices ($\rho_{vc/cv}$) and diagonal wave vector dependence ($\rho^{kk/kk^{\prime \prime }}$). Due to those properties, we found that direct matrix elements are generally complex numbers, while exchange matrix elements are real. Additionally, due to dependence only on the diagonal wave vector, the exchange matrix element can be computed much faster than the direct matrix elements. At this point, we note also that at first glance there is $\vec{G}=0$ singularity in $V^{X}$. This singularity is generally problematic for 3D crystals and different methods of dealing with it have been discussed in the literature [329]. However, for a 2D crystal, the singular term is equal to 0 and can be excluded from the summation over G vectors in Equation (16).

We note that the Bethe–Salpeter equation defined in Equation (11) is gauge-invariant, i.e., it does not depend on the gauge choice of wave functions, which can be created arbitrarily for every band and *k* point. On the other hand, the numerical phase affects the phase of the exciton coefficients. This arbitrariness of phase choice may influence the apparent symmetry of the excitonic ground state [253]. One of the solutions to this problem is to study only observable quantities, such as the imaginary part of the dielectric function $\varepsilon_{2}$, in which complex exciton amplitudes are multiplied by velocity operator matrix elements, which are also constructed from wave functions. Keeping track of the phase in both should be enough to obtain a gauge-independent answer. However, sometimes one is interested in studying excitonic wave functions themselves. To obtain ground excitonic states with 1s symmetry, various numerical approaches are used; however, their details are rarely discussed in the literature [200,253]. In our procedure, we follow the idea introduced by Rohlfing and Louie [200], in which the global phase of tight-binding wave functions is chosen in such a way that the sum of imaginary parts is 0, i.e., ∑α=12∑μ=13Imvαμ(n)=0. In the next step, we rotate the global phase to obtain phase 0 in the $m{}_{d}=0$ orbital, which means that the phase of the second TB coefficient is set to zero (Imv12=0). The second step breaks the first property, although we found that it is necessary to numerically obtain excitons in the +K and −K valleys, the wave functions of which have $A_{n}\left(-k\right)=A_{n}^{*}\left(k\right)$ properties, as expected from time-reversal symmetry arguments.

Just as for the exchange Coulomb interaction discussed above, it is easy to understand that the diagonal term of the direct electron–hole interaction in BSE given by Equation (11) is singular at $k=k^{\prime }$ and G=0. Renormalization due to this singularity has to be included in simulations on the finite lattice, otherwise numerical results give vastly different results when compared with theoretical predictions. Dealing with this singularity is connected with the discretization of the BZ associated with a single valley. Below, we discuss the rectangular discretization of the lattice. Confining our discussion to the case of G=0 for a moment, one of the methods that allow to integrate out the singularity is to note that form factor $F^{D}\left(k=k^{\prime },G=0\right)=1$ and make an approximation that the exciton wave function inside the box centered around the point ($k_{x}$,$k_{y}$) takes a constant value. This approximation is naturally more and more exact with an increasing number of points (and a decreasing area associated with each *k* point), into which the BZ is discretized. This allows us to approximate the diagonal of the BSE interaction kernel as

(18) $$\int_{k_{x}-\delta k/2}^{k_{x}+\delta k/2}\int_{k_{y}-\delta k/2}^{k_{y}+\delta k/2}dk_{x}^{\prime }dk_{y}^{\prime }\frac{A_{n}\left(\vec{k^{\prime }}\right)}{\left|\vec{k}-\vec{k^{\prime }}\right|}\approx A_{n}\left(\vec{k}\right)V_{\sin.}\delta k.$$

We note that this result assumes a constant, static screening of the electron–hole interaction and in principle should be recalculated when the *k*-dependent screening model is used. Additionally, the G=0 term is the leading one; however, summation over *G* vectors introduces further corrections into the singular term. We checked numerically that both effects introduce contributions that are at least two orders of magnitude smaller than the expression given in Equation (18) and are neglected in further studies. We note that the constant $V_{sin.}\approx 3.5255$ is calculated for the rectangular lattice with an area of BZ around the single point given by ${\left(\delta k\right)}^{2}$, and it should be changed to $V_{sin.}\approx 3.2325$ for a rhomboidal lattice with an analogous rhombus area given by $\sqrt{3}{\left(\delta k\right)}^{2}/2$ under the same approximations as for the rectangular discretization scheme.

In the next step, we build a systematic theory of approximation for the direct electron–hole matrix elements. The calculation of the interaction form factors $F^{D}$ is a major bottleneck of both ab initio and TB calculations due to the necessity of calculating them for all combinations of *k* and $k^{\prime }$, and the additional summation over reciprocal lattice vectors *G*. The easiest solution is to assume all form factors to be one (their highest possible value, exact for $k=k^{\prime }$ and G=0) and note that the highest value-entering sum over *G* vectors comes from *G*s minimizing $|k-k^{\prime }-G|$ distance. We checked that this approximation is useful around the *K* point, for which the neighborhood-form factor absolute value deviates from one rather slowly. The approximation described above allows us to reproduce numerically analytical solutions for a simple model of the exciton with an electron/hole dispersion in a parabolic approximation. Its main deficiency is that it does not include any effects related to the orbital composition of the bands (no Bloch function effect) and is purely real, which always gives degenerate states in the exciton spectrum with the same exciton angular momentum quantum numbers. To motivate a way around this problem, let us first discuss which parts of the full, complex form factor influence mainly its value. For concreteness, let us discuss, for example, the difference between a form factor describing the scattering between one chosen *k* point to some $k^{\prime }+\Delta k^{\prime }$ point ($\Delta k^{\prime }$ is assumed to be small). One can quickly deduce that the effect of $\Delta \vec{k^{\prime }}$ on the matrix element $V^{D}$ is complicated: it affects (1) the denominator $1/|k-k^{\prime }-G|$; (2) tight-binding coefficients inside $\rho_{vv/cc}$; (3) exponent values depending on z, z′; and (4) details of the in-plane integration of Slater orbitals and the summation over unit cells. We checked numerically, implementing in code the selective turning on/off of all the above contributions so that, actually, the first two corrections (denominator and TB coefficient) yield a very good approximation to the matrix elements and the last two (exponent and details of integration) do not contribute too much. This motivated us to extract TB coefficients from form factors, giving an expression formally equivalent to Equation (14) with an implicit summation over sublattices (α,β) and orbitals (μ,ν), which can be written as

(19) $$F^{D}\left(\vec{k},\vec{k^{\prime }},\vec{G}\right)=\sum_{\alpha \beta \alpha^{\prime }\beta^{\prime }=1}^{2}\sum_{\mu \nu \mu^{\prime }\nu^{\prime }=1}^{3}{\left[v_{\alpha \mu }^{VB}\left(\vec{k^{\prime }}\right)\right]}^{*}v_{\beta \nu }^{VB}\left(\vec{k}\right){\left[v_{\alpha^{\prime }\mu^{\prime }}^{CB}\left(\vec{k}\right)\right]}^{*}v_{\beta^{\prime }\nu^{\prime }}^{CB}\left(\vec{k^{\prime }}\right)F_{\alpha \beta \alpha^{\prime }\beta^{\prime }\mu \nu \mu^{\prime }\nu^{\prime }}\left(\vec{k},\vec{k^{\prime }},\vec{G}\right),$$

and the quantity we call “orbital form factor” $F_{\alpha \beta ...}^{D}$ is given by

(20) $$F_{\alpha \beta \alpha^{\prime }\beta^{\prime }\mu \nu \mu^{\prime }\nu^{\prime }}\left(\vec{k},\vec{k^{\prime }},\vec{G}\right)=\int dz\int dz^{\prime }{\tilde{\rho }}_{\alpha \beta \mu \nu }^{\vec{k^{\prime }}\vec{k}}\left(\vec{G},z\right){\tilde{\rho }}_{\alpha^{\prime }\beta^{\prime }\mu^{\prime }\nu^{\prime }}^{\vec{k}\vec{k^{\prime }}}\left(-\vec{G},z^{\prime }\right)e^{-\left|z-z^{\prime }\right|\cdot \left|\vec{k^{\prime }}-\vec{k}-\vec{G}\right|}.$$

Those orbital form factors depend on analogues of product densities, now in the form that does not depend on TB coefficients. This method, being equivalent to Equation (14), is not faster; however, it helps to realize that the $C^{TB}$ coefficient can be calculated very fast from the TB model and we can take only the orbital form factor at the $k^{\prime }-k-G=0$ limit. This approximation is conceptually equivalent to treating the long-range Coulomb interaction as not dependent on the details of the orbital structure and taking the maximal absolute value of the orbital form factor (=1). Interestingly, taking this limit also produces a phase $\phi =\vec{G}\cdot \left(-\tau_{\alpha }+\tau_{\alpha^{\prime }}\right)$, related to non-zero *G* and the position of atoms inside unit cell:

(21) $$F_{\alpha \beta \alpha^{\prime }\beta^{\prime }\mu \nu \mu^{\prime }\nu^{\prime }}\left(\vec{k}-\vec{k^{\prime }}-\vec{G}=0\right)=\delta_{\alpha \beta }\delta_{\alpha^{\prime }\beta^{\prime }}\delta_{\mu \nu }\delta_{\mu^{\prime }\nu^{\prime }}e^{i\vec{G}\left(-{\vec{\tau }}_{\alpha }+{\vec{\tau }}_{\alpha^{\prime }}\right)}.$$

The final equation for the direct form factor is, therefore, simplified to

(22) $$F^{D}\left(\vec{k},\vec{k^{\prime }},\vec{G}\right)=\sum_{\alpha \alpha^{\prime }=1}^{2}\sum_{\mu \mu^{\prime }=1}^{3}{\left[v_{\alpha \mu }^{VB}\left(\vec{k^{\prime }}\right)\right]}^{*}v_{\alpha \nu }^{VB}\left(\vec{k}\right){\left[v_{\alpha^{\prime }\mu^{\prime }}^{CB}\left(\vec{k}\right)\right]}^{*}v_{\alpha^{\prime }\mu^{\prime }}^{CB}\left(\vec{k^{\prime }}\right)e^{i\vec{G}\left(-{\vec{\tau }}_{\alpha }+{\vec{\tau }}_{\alpha^{\prime }}\right)}.$$

This expression is an extension of results presented in Refs. [245,247,250,330] to include summation over non-zero *G* vectors. We note that the phase rotation presented in Equation (22) is either 1 or $C_{3}$ rotation equal to $\exp\left(\pm 2\pi i/3\right)$.

As is known from the literature and from our own experience, the computation of the excitonic spectrum is a numerically challenging task. To lower computational complexity even further, let us discuss now how excitonic fine-structure calculations are performed in practice. First, we discretize Equation (11), neglecting the electron–hole exchange interaction, since it is much weaker than the direct electron–hole interaction. Additionally, we choose in summation over $k^{\prime }$ wave vectors only those associated with one valley, as shown in Figure 7. Remembering about singular terms as discussed previously, the Bethe–Salpeter-like equation for one valley takes the form

(23) $$\left[\Delta E\left(\vec{k}\right)-\Delta_{\mathrm{GAP}}-V_{\sin.}\right]A_{n}\left(\vec{k}\right)-\sum_{\vec{k^{\prime }}\neq \vec{k}}^{1/2\mathrm{BZ}}{\left(\delta k\right)}^{2}V^{D}\left(\vec{k},\vec{k^{\prime }}\right)A_{n}\left(\vec{k^{\prime }}\right)=E_{n}A_{n}\left(\vec{k}\right).$$

This equation represents a dense, Hermitian matrix that is diagonalized numerically. The primary convergence parameter is the number of *k* vectors into which a single valley was discretized. Details of computations and convergence studies are presented in our works [281,282].

A further step in fine-structure calculations is to add spin splitting to both valence and conduction bands. Spin-splitting modifies the electron–hole energy difference $\Delta E\left(\vec{k}\right)=\varepsilon_{\mathrm{CB}}^{\sigma }\left(\vec{k}\right)-\varepsilon_{\mathrm{VB}}^{\sigma^{\prime }}\left(\vec{k}\right)$. To calculate matrix elements, we choose to use spinless wave functions due to their negligible dependence on spin. By this method, we are able to obtain a fine structure in one valley, i.e., both bright and dark A and B excitonic series. The calculation of fine structure in one valley automatically gives fine structure in the other one due to the symmetry of energies $E_{n}\left(+K\right)=E_{n}\left(-K\right)$ for spin-flipped configurations of excitons. We also found that with a proper gauge of matrix elements, the $V\left(-\vec{k},-{\vec{k}}^{\prime }\right)=V\left({\vec{k}}^{\prime },\vec{k}\right)$ property of the matrix elements between valleys is satisfied. Implementing this symmetry in BSE, one can formally prove that exciton wave functions have to be related to each other as $A_{n}^{*}\left(-\vec{k}\right)=A_{n}\left(\vec{k}\right)$. We checked numerically, performing separate, full calculations in +K and −K valleys that our implementation satisfies these properties, giving within numerical precision the same excitonic spectrum and phases of excitonic states related by the mirror symmetry of *k* vector and complex conjugation.

### 3.3. Screening of Coulomb Interactions

An additional complication in the realistic description of the Coulomb electron–hole interaction stems from the screening of the interaction by other carriers. As noted early in studies of graphene [331], electron–electron interaction screening in 2D crystals behaves differently than in 3D crystals. Therefore, it is necessary to use a more involved screening model. The most well known model that captures the major linear dependence of screening in *k*-space was derived in the physics of thin dielectric slabs and is called the Rytova–Keldysh (R.-K.) model [272,273,331]. This approximation has been used in numerous works [53,260,265,269,270,271,331,332]. In this theory, the bare Coulomb matrix elements $V^{D/X}$ are divided by a dielectric function

(24) $$V_{R\text{-}K}^{D/X}\left(q\right)=\frac{V_{bare}^{D/X}\left(q\right)}{\varepsilon_{r}^{R\text{-}K}\left(1+2\pi \alpha \left|\vec{q}\right|\right)}.$$

**Figure 10:**
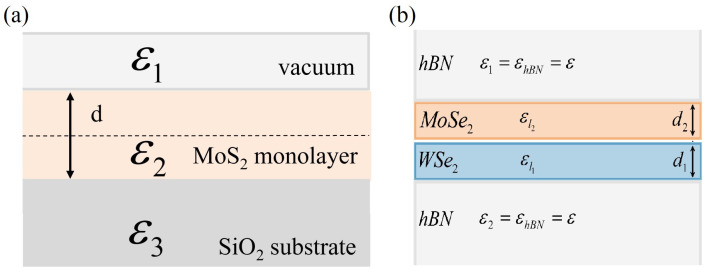
(**a**) Schematic picture of the dielectric environment of the MoS${}_{2}$ monolayer on the SiO${}_{2}$ substrate. (**b**) Slab model of the MoSe${}_{2}$/WSe${}_{2}$ heterostructure. Each of the MX${}_{2}$ layers with the width $d_{1,2}$, respectively, is described by the dielectric constant $\varepsilon_{l_{1},l_{2}}$.

Counterintuitively, the static screening part $\varepsilon_{r}$ depends not on the material itself, but on the surrounding material’s dielectric properties $\varepsilon_{r}^{R\text{-}K}=\left(\varepsilon_{1}+\varepsilon_{3}\right)/2$. This expression comes from the theory of a dielectric slab with a finite width *d* surrounded by two semi-infinite dielectrics with electric permeability $\varepsilon_{1},\varepsilon_{3}$. In the numerical results for MX${}_{2}$ discussed in the next sections, we consider two specific cases of the dielectric environment. In the first step, we analyze MoS${}_{2}$ (with “effective” thickness d=6.14 Å) on top of the bulk SiO${}_{2}$ ($\varepsilon_{3}=4$) crystal [97,333], see Figure 10a for reference. We assume that from the top, the monolayer is surrounded by a vacuum; therefore, we take $\varepsilon_{3}=1$. Next, we consider the case of both MoS${}_{2}$ and MoSe${}_{2}$ encapsulated by hBN (as shown in Figure 10b), where we take $\varepsilon_{1}=\varepsilon_{3}=4.5$ [54].

As is known from the literature [334], more advanced models of screening do not significantly affect exciton spectra. This is related to the property that the R.-K. model describes well—screening from ab initio and only for large *k*-space distance (when matrix elements are small due to $1/|k-k^{\prime }|$ dependence) there is substantial difference between “correct” and approximated screening models.

The realistic description of the Coulomb electron–hole interaction becomes even more complicated in the case of heterostructures [278,280,335,336,337]. Furthermore, the complex form of the system leads to different formulas of dielectric functions for intra- and interlayer electron–electron interactions [335]. The Coulomb interaction potential for MX${}_{2}$ bi-layers can be written in the form analogous to Equation (24) as

(25) $$V^{\mathrm{inter}/\mathrm{intra}}\left(\vec{q}\right)=\frac{V_{\mathrm{bare}}\left(q\right)}{\varepsilon_{r}^{l_{i},l_{j}}\left(\vec{q}\right)}.$$

Following Ref. [335], analysing the result of Poisson’s equation in the limit of qd<<1, we can rewrite $\varepsilon^{l_{i},l_{j}}$ as

(26) $$\varepsilon^{l_{1},l_{1}}\left(q\right)\approx \varepsilon^{l_{2},l_{2}}\left(q\right)=\varepsilon_{r}^{R\text{-}K}\left[1+\alpha_{R\text{-}K}^{intra}\cdot q\right]$$

and

(27) $$\varepsilon^{l_{1},l_{2}}\left(q\right)\approx \varepsilon_{r}^{R\text{-}K}\left[1+\alpha_{R\text{-}K}^{inter}\cdot q\right].$$

In the above, R.-K. screening formulas $\alpha_{R\text{-}K}^{intra}$ and $\alpha_{R\text{-}K}^{inter}$ can be treated as an effective polarizability estimated from experimentally known 1s-state energies of both intra- and interlayer excitons. One can expect that the interlayer screening model leads to the renormalization of the exciton spectrum, similarly to the monolayer case [281]. Interestingly, the interlayer R.-K. model gives a more precise dielectric screening description compared to the monolayer case [335]. We note that when we discuss the numerical results for the MoSe${}_{2}$/WSe${}_{2}$ heterostructure encapsulated by hBN, we use $\varepsilon_{1}=\varepsilon_{3}=4.5$ [54].

## 4. Mechanisms of the Renormalization of the X Spectrum

In the following section, several mechanisms affecting excitonic fine structures are briefly discussed, with in-depth discussion already presented elsewhere [281]. Importantly, we note that our main motivation to construct the presented theory was understanding the effects of the multivalley (*K* and *Q* points) band structure of MX${}_{2}$ and the topology of the wave functions on the excitonic fine structure. Instead of focusing on agreement with ab initio + GW + Bethe–Salpeter, we note that our approach offers greater tunability, allowing to understand various physical mechanisms (e.g., role of dispersion, effective mass, strength of interactions, topology of wave functions, and screening mechanisms, etc.) contributing to the complicated problem of the excitonic spectrum. The understanding of these aspects is necessary to build a theory of optical response, e.g., in quantum dots, twisted bi-layer systems, or in the presence of magnetic fields, problems that are usually not easy to solve in the ab initio + GW + Bethe–Salpeter framework. We start with the effect of the band structure on excitonic levels. How different approximations to conduction and valence band energies affect the electron–hole pair energy is summarized in Figure 6. Direct transition energies enter as diagonal to the Bethe–Salpeter equation. In the parabolic-effective mass approximation with simplified bare Coulomb interactions and static screening, the excitonic spectrum gives the binding energy of the first 1s state equal to −4 Ry${}^{\mu }$ and the degenerate second shell of three states: 2s, $2p_{x}$, $2p_{y}$, whose energies are $E_{n=2-4}=-4/9$ Ry${}^{\mu }$. When we change the dispersion model to a massive Dirac fermion, keeping interaction and screening as previously, we notice that the binding energy of the 1s state lowers to ≈−5.5 Ry${}^{\mu }$. This behavior can be understood as an increased “average” effective mass, i.e., carriers in the massive Dirac fermion model on average taken over the BZ are described better by a higher effective mass than in the parabolic model. This naturally leads to stronger binding, corresponding to a lower energy of the 1s state. We also observe a larger separation between the first and second shells and a small breaking of degeneracy between 2s and $2p_{x},2p_{y}$ states within the second shell. Finally, when a tight-binding dispersion model is used, the effects described for the massive DF model become even more pronounced. The energy of the 1s state lowers as much as to −10 Ry${}^{\mu }$, there is large renormalization of the 1s–2s states’ energy differences, and states with exciton angular momentum L=0 (2s) and |L|=1 are no longer degenerate. A similar effect is observed for higher shells. In addition to the “average lowering” of the effective mass process, there is a pronounced contribution coming from the existence of *Q* points that can be observed in excitonic wave functions. The breaking of the degeneracy of the 3s and 4s state for L≠0 is also observed, together with the breaking of the degeneracy of states with different *L*. We conclude that due to existence of three *Q* points around *K* point in the single valley, the full rotational symmetry of *s*-like states is broken and therefore those states react more strongly than others to changes in electron–hole energy dispersion from the parabolic to the TB.

As discussed in the previous section, constant screening does not faithfully describe the actual screening of interactions in 2D semiconductors. The simplest correction, capturing the non-locality of screening via its $|q-q^{\prime }|$ dependence can be modeled by Rytova–Keldysh theory and parametrized by polarizability α. The excitonic spectrum is heavily renormalized for TB model dispersion and the $1/|q-q^{\prime }|$ interaction when static screening is switched from the homogeneous one to the R.-K. model. Non-local screening has an opposite effect than changing dispersion from parabolic to TB, and 1s state energy rises from −10 Ry${}^{\mu }$ back to approximately −4 Ry${}^{\mu }$. Additionally, split L=0 and L≠0 excited exciton states drastically change their energies, reversing the order of arrangement of excitonic states.

**Figure 11:**
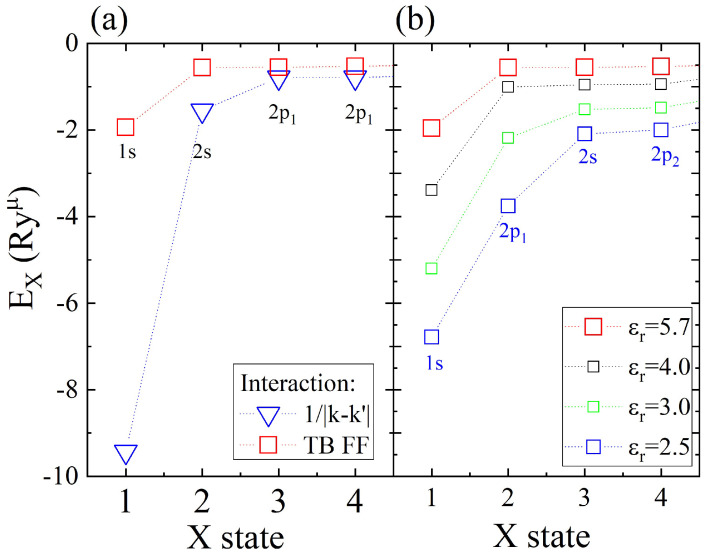
The first two shells of the excitonic spectrum with full tight-binding direct interaction form factors. (**a**) Effect of the form factors compared to $F^{D}=1$ approximation. (**b**) Effect of different static screening on the spectrum. On both (**a**,**b**), tight-binding energies of electron and hole are used.

In addition to the effects of dispersion and screening that renormalizes the 2D exciton spectrum, the direct electron–hole interaction form factors $F^{D}$ have to be taken into account. Focusing on complex values of the form factors, the result of a calculation on a 7000 *k*-point grid (the largest we were able to study with the full effect of wave functions) that allows for a reliable discussion of the first and second excitonic shell is presented in Figure 11. Generally speaking, because its value is ≤1, its averaged effect translates into lowering the value of binding energy and pushing excitonic shells toward each other, as shown in Figure 11a. Due to the strong effect of tight-binding wave functions, it is understood that with realistic form factors, different parameters of screening have to be chosen, for example, as shown for static screening in Figure 11b. We conclude that the collective effect of the renormalization of the 2D Rydberg series by dispersion, non-local interaction screening, and carrier wave functions is to make s-like series look like a “more than 3D” exciton. It means that even though the exciton is physically confined to the 2D plane of MX${}_{2}$, its excited state series resembles more the 3D excitonic Rydberg ladder of states. We note that non-hydrogenic Rydberg series is usually explained as an effect of non-local screening [47]. We find that it is not only related to screening, but also depends heavily on dispersion, especially secondary minima in the CB at *Q* points, and wave function contributions affecting the electron–hole interaction.

As mentioned before, taking into account electron and hole wave functions affects the exciton fine structure by contributing to the renormalization of s-like states. In addition to that, another interesting effect occurs. Because the direct electron–hole interaction depends on wave functions, its value is in general complex and phases of matrix elements $V^{D}\left(\vec{k},{\vec{k}}^{\prime }\right)$ cannot be set to zero by any gauge transformation. The role of a complex interaction phase manifests itself in two effects: first, it slightly renormalizes positions of *s*-like states, however, this effect is small. On the other hand, a clearly visible effect is that in the second shell, normally degenerate p-like states become split in energy and mix, forming $2p_{\pm 1}=2p_{x}\pm i2p_{y}$ states. More generally, due to complex interactions, all states with a non-zero exciton momentum L also mix and split (e.g., $3p_{\pm }$, $3d_{\pm }$, etc.).

The splitting of states with non-zero L reminds of the situation when a magnetic field is applied. In our case there is no magnetic field but instead there is a “geometric” field resulting from the topology of wave functions. This orbital moment, described by the gauge invariant Berry connection, has to come from the properties of wave functions. How to trace this Berry’s connection effect on the electron–hole interaction has been shown elsewhere [282,338,339].

Now, let us focus for a moment on the effect of spin splitting on the exciton fine structure. The largest effect, related to the splitting of bands in the VB, is A-B exciton splitting. For $\Delta_{VB}^{SOC}=148$ meV we obtain A-B *1s* excitonic states split by ≈125 meV for α=0.5 and the full TB model. A much more subtle effect, introducing the splitting of the A exciton state to spin bright (same spin arrangement in the VB and CB) and spin dark (opposite spins) is related to interplay between spin splitting in the CB and the different dispersion of bands, which can be understood approximately as different effective masses for carriers with different spins. Even though the single-particle arrangements of bands point to a “bright smaller than dark” arrangement, different effective masses cause an inversion of excitonic states and the spin-dark state becomes a ground excitonic state. We conclude that this situation happens in MoS${}_{2}$, which has very small $\Delta_{CB}^{SOC}\approx 3$ meV. This conclusion is consistent with recent GW-BSE calculations [219,230] and some experiments [67].

Up to this point, we neglected generally small electron–hole exchange interactions. This interaction, however, controls the splitting of dark and bright states, because it affects only excitons built from an electron and hole with the same spin. It increases the energy of the exciton as a result of quantum mechanical electron–hole repulsion, in opposite to direct interaction. Starting with spin-degenerate states to make our analysis more transparent, when no exchange interaction $V^{X}$ is present, dark and bright states are degenerate. When an unscreened electron–hole interaction is turned on, the bright-state energy increases (the binding energy is lowered) up to ≈20 meV, depending on the choice and details of the screening model. When homogenous screening or R.-K. models are applied also to exchange interactions, this value is significantly lowered. We conclude that in all cases, the trend is such that the dark excitonic state in MoS${}_{2}$ has the lowest energy, adding up to a similar conclusion from the spin splitting discussion above. We note that in the DFT + GW + Bethe–Salpeter approach [219], the electron–hole exchange interaction is always taken as the bare, unscreened one. In our approach, derived from the CI approach, both direct and exchange interactions are treated on an equal footing and should be screened in the same way. This issue has been discussed in the literature [230,325], but in our view further studies are necessary to understand the source of this discrepancy. On the other hand, irrespective to details of screening, we conclude that in MoS${}_{2}$, the lowest in the energy excitonic state should be dark due to spin and exchange effects.

In next step, we analyze the excitonic spectrum of the MoS${}_{2}$ monolayer using the combination of the minimal tight-binding model, Bethe–Salpeter equation, and simplified Coulomb interactions theory, with form factors given by Equation (22) and the Rytova–Keldysh screening model. The exciton fine structure has been determined in two cases, namely the MoS${}_{2}$ monolayer on the SiO${}_{2}$ substrate and the hBN encapsulated MoS${}_{2}$, respectively. We restrict ourselves to the first three shells (n=1,2,3). For MoS${}_{2}$ on the SiO${}_{2}$ and MoS${}_{2}$ encapsulated in hBN we set the $\alpha_{Keldysh}$ polarizability to correctly determine the experimentally known exciton ground state [54,247]. The *1s*-state has been taken as $E_{n=1}^{SiO_{2}}=-0.335$ eV and $E_{n=1}^{hBN}=-0.231$ eV, so that we obtained $\alpha_{Keldysh}=2.0$ for the SiO${}_{2}$ substrate and $\alpha_{Keldysh}=0.75$ for the hBN surrounding.

**Figure 12:**
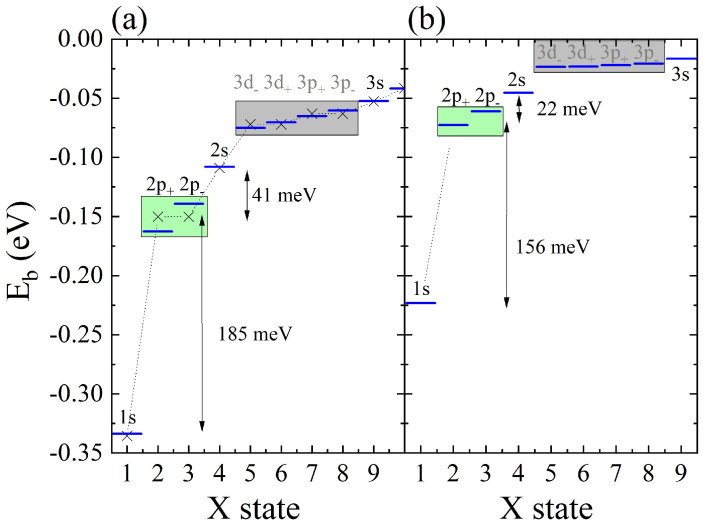
Exciton fine structure for the MoS${}_{2}$ monolayer (**a**) on the SiO${}_{2}$ substrate and (**b**) encapsulated with hBN, in a full TB model with complex electron–hole interaction included. Results restricted to the first three shells. The topological splitting of 2p, 3p, and 3d states in the excitonic spectrum of the MoS${}_{2}$ layer is presented.

Figure 12 shows the excitonic spectrum determined for the MoS${}_{2}$ monolayer. We restricted ourselves to n=1,2,3 (first three shells) only. For both types of surroundings, the 2s state is characterized by a smaller binding energy than the 2p states. Moreover, we deal with generic topological effects manifested in the exciton spectrum resulting from the properties of electron and hole Bloch wave functions, especially the Berry curvature. Inclusion of the effect of wave functions on the exciton spectrum results in the topological splitting of 2p, 3p, and 3d states.

The excitonic spectrum for the MoS${}_{2}$ monolayer on the SiO${}_{2}$ substrate, shown in Figure 12a, is characterized by the exciton binding energy $E_{b}=-335$ meV. The energy splitting between the *1s* and *2s* states has been determined to be $E^{1s-2s}=226$ meV. In the hBN-encapsulated MoS${}_{2}$ case presented in Figure 12b, both the excitonic binding energy $E_{b}=-223$ meV and the determined 1s–2s energy splitting $E^{1s-2s}=176$ meV are smaller than for the MoS${}_{2}$ on SiO${}_{2}$.

**Figure 13:**
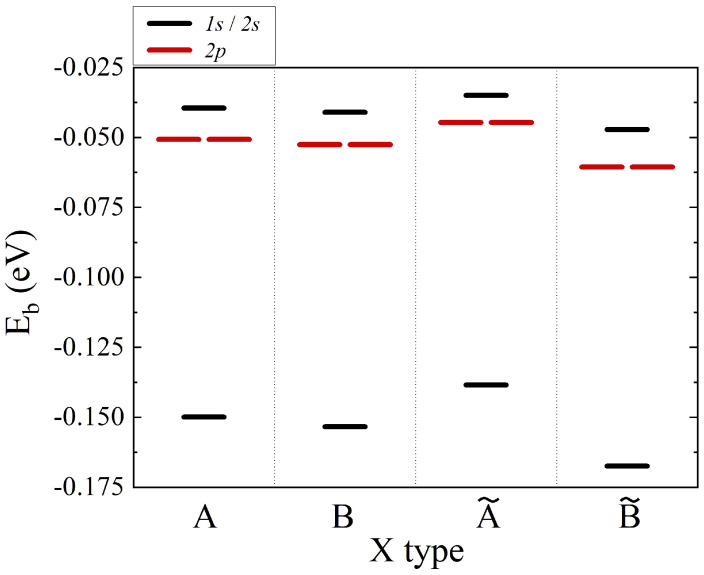
Interlayer exciton fine structure for the MoSe${}_{2}$/WSe${}_{2}$ heterostructure encapsulated with hBN in the EMA, restricted to 1s, 2p, and 2s states. Interlayer *A/B/$\tilde{A}$/$\tilde{B}$* exciton types have been distinguished, where *A* denotes the transition WSe${}_{2}$–MoSe${}_{2}$ spin-up, *B* transition MoSe${}_{2}$–WSe${}_{2}$ spin-down, $\tilde{A}$ transition MoSe${}_{2}$–WSe${}_{2}$ spin-up, and $\tilde{B}$ transition WSe${}_{2}$–MoSe${}_{2}$ spin-down, respectively.

We now turn to the effect of the second atomic layer on the exciton spectrum. Using the combination of effective mass approximation and the Bethe–Salpeter equation, including the interlayer Rytova–Keldysh screening [335], we now analyze the interlayer exciton spectrum for MoSe${}_{2}$/WSe${}_{2}$ encapsulated in hBN [340]. For various spin-split combinations of bands, we study the fine structure for excitons with zero total momentum $Q^{X}=0$ (at *K* point). Excitonic states built from electrons and holes from distinct layers present a rich spectrum of different types of optical transitions. In Figure 13 we consider interlayer *A/B/$\tilde{A}$/$\tilde{B}$* exciton series of MoSe${}_{2}$/WSe${}_{2}$, where *A* denotes the transition between WSe${}_{2}$ and MoSe${}_{2}$ spin-up, *B* transition MoSe${}_{2}$–WSe${}_{2}$ spin-down, $\tilde{A}$ transition MoSe${}_{2}$–WSe${}_{2}$ spin-up, and $\tilde{B}$ transition WSe${}_{2}$–MoSe${}_{2}$ spin-down, respectively. We restrict ourselves to 1s, 2p, and 2s states. The parameter $\alpha_{RK}^{inter}$ was set in order to correctly determine the experimentally known *1s*-state interlayer exciton energies [142]. We take the ground state binding energy of the interlayer exciton as $E_{n=1}^{B}=-150$ meV [142] and obtain $\alpha_{RK}^{inter}=2.30$.

In Figure 13, we show the excitonic spectrum of the MoSe${}_{2}$/WSe${}_{2}$ heterostructure, restricted to *1s*, *2p*, and *2s* states only. Considering all interlayer exciton types, namely *A/B/$\tilde{A}$/$\tilde{B}$* excitons, we can analyze both the *A-B / $\tilde{A}$-$\tilde{B}$* and 1s–2s splittings. Interestingly, the values for all interlayer *1s-2s* splittings have been determined as Δ(1s−2s)≈10 meV, while Δ(A−B)≈50 meV and $\Delta (\tilde{A}-\tilde{B})\approx 30$ meV. Including in our studies various spin-split combinations of bands, considering not only the energetically lowest optical transitions (*A* excitons), we predict rich interlayer exciton series resulting with additional peaks due to the presence of all *A/B/$\tilde{A}$/$\tilde{B}$* exciton types. More work is needed to understand intra- and interlayer excitons in 2D heterostructures.

## 5. Summary

In summary, in this review we describe the excitonic problem in transition metal dichalcogenide semiconductors. We discussed building an ab inito-based tight-binding model that captures all the important features of the electronic structures of these materials. Then, we describe our theory of exciton, focusing on issues related to the evaluation of the interaction matrix elements. Then, we presented how such a theory can be used to understand the physics of the excitonic spectrum in both mono- and bi-layer heterostructures. We discussed the effect of band nesting on the exciton fine structure; Coulomb interactions; and the topology of wave functions, screening, and the dielectric environment. Finally, we followed by adding another layer and discussed excitons in heterostructures built from two-dimensional semiconductors. We hope that this review will be helpful to people who are interested in entering the fascinating field of the optical properties of low-dimensional semiconductors.

## Data Availability

Not applicable.

## Abbreviations

The following abbreviations are used in this manuscript:

TMDs	Transition metal dichalcogenides
X	Exciton
hBN	Hexagonal boron nitride
BSE	Bethe–Salpeter equation
VB	Valence band
CB	Conduction band
DFT	Density functional theory
SOC	Spin-orbit coupling
TB	Tight-binding
BZ	Brillouin zone
EMA	Effective mass approximation
mDF	Massive Dirac fermion
R.-K.	Rytova–Keldysh

## References

[1] Wallace P.R. (1947). The Band Theory of Graphite. Phys. Rev..

[2] Connell G., Wilson J., Yoffe A. (1969). Effects of pressure and temperature on exciton absorption and band structure of layer crystals: Molybdenum disulphide. J. Phys. Chem. Solids.

[3] Wilson J., Yoffe A. (1969). The transition metal dichalcogenides discussion and interpretation of the observed optical, electrical and structural properties. Adv. Phys..

[4] Novoselov K.S., Geim A.K., Morozov S.V., Jiang D., Zhang Y., Dubonos S.V., Grigorieva I.V., Firsov A.A. (2004). Electric Field Effect in Atomically Thin Carbon Films. Science.

[5] Novoselov K.S., Geim A.K., Morozov S.V., Jiang D., Katsnelson M.I., Grigorieva I.V., Dubonos S.V., Firsov A.A. (2005). Two-dimensional gas of massless Dirac fermions in graphene. Nature.

[6] Zhang Y., Tan Y.W., Stormer H.L., Kim P. (2005). Experimental observation of the quantum Hall effect and Berry’s phase in graphene. Nature.

[7] Novoselov K.S., Jiang D., Schedin F., Booth T.J., Khotkevich V.V., Morozov S.V., Geim A.K. (2005). Two-dimensional atomic crystals. Proc. Natl. Acad. Sci. USA.

[8] Mounet N., Gibertini M., Schwaller P., Campi D., Merkys A., Marrazzo A., Sohier T., Castelli I.E., Cepellotti A., Pizzi G. (2018). Two-dimensional materials from high-throughput computational exfoliation of experimentally known compounds. Nat. Nanotechnol..

[9] Chhowalla M., Shin H.S., Eda G., Li L.J., Loh K.P., Zhang H. (2013). The chemistry of two-dimensional layered transition metal dichalcogenide nanosheets. Nat. Chem..

[10] Duan X., Wang C., Pan A., Yu R., Duan X. (2015). Two-dimensional transition metal dichalcogenides as atomically thin semiconductors: Opportunities and challenges. Chem. Soc. Rev..

[11] Wang Q.H., Kalantar-Zadeh K., Kis A., Coleman J.N., Strano M.S. (2012). Electronics and optoelectronics of two-dimensional transition metal dichalcogenides. Nat. Nanotechnol..

[12] Lien D.H., Kang J.S., Amani M., Chen K., Tosun M., Wang H.P., Roy T., Eggleston M.S., Wu M.C., Dubey M. (2015). Engineering Light Outcoupling in 2D Materials. Nano Lett..

[13] Lin Y., Ling X., Yu L., Huang S., Hsu A.L., Lee Y.H., Kong J., Dresselhaus M.S., Palacios T. (2014). Dielectric Screening of Excitons and Trions in Single-Layer MoS_2_. Nano Lett..

[14] Raja A., Chaves A., Yu J., Arefe G., Hill H.M., Rigosi A.F., Berkelbach T.C., Nagler P., Schüller C., Korn T. (2017). Coulomb engineering of the bandgap and excitons in two-dimensional materials. Nat. Commun..

[15] Ajayi O.A., Ardelean J.V., Shepard G.D., Wang J., Antony A., Taniguchi T., Watanabe K., Heinz T.F., Strauf S., Zhu X.Y. (2017). Approaching the intrinsic photoluminescence linewidth in transition metal dichalcogenide monolayers. 2D Mater..

[16] Cadiz F., Courtade E., Robert C., Wang G., Shen Y., Cai H., Taniguchi T., Watanabe K., Carrere H., Lagarde D. (2017). Excitonic Linewidth Approaching the Homogeneous Limit in *MoS*_2_-Based van der Waals Heterostructures. Phys. Rev. X.

[17] Splendiani A., Sun L., Zhang Y., Li T., Kim J., Chim C.Y., Galli G., Wang F. (2010). Emerging Photoluminescence in Monolayer MoS_2_. Nano Lett..

[18] Mak K.F., Lee C., Hone J., Shan J., Heinz T.F. (2010). Atomically Thin *MoS*_2_: A New Direct-Gap Semiconductor. Phys. Rev. Lett..

[19] Eda G., Yamaguchi H., Voiry D., Fujita T., Chen M., Chhowalla M. (2011). Photoluminescence from Chemically Exfoliated MoS_2_. Nano Lett..

[20] Tonndorf P., Schmidt R., Böttger P., Zhang X., Börner J., Liebig A., Albrecht M., Kloc C., Gordan O., Zahn D.R.T. (2013). Photoluminescence emission and Raman response of monolayer MoS_2_, MoSe_2_, and WSe_2_. Opt. Express.

[21] Zhao W., Ghorannevis Z., Chu L., Toh M., Kloc C., Tan P.H., Eda G. (2013). Evolution of Electronic Structure in Atomically Thin Sheets of WS_2_ and WSe_2_. ACS Nano.

[22] Ruppert C., Aslan O.B., Heinz T.F. (2014). Optical Properties and Band Gap of Single- and Few-Layer MoTe_2_ Crystals. Nano Lett..

[23] Lezama I.G., Arora A., Ubaldini A., Barreteau C., Giannini E., Potemski M., Morpurgo A.F. (2015). Indirect-to-Direct Band Gap Crossover in Few-Layer MoTe_2_. Nano Lett..

[24] Kadantsev E.S., Hawrylak P. (2012). Electronic structure of a single MoS_2_ monolayer. Solid State Commun..

[25] Ramasubramaniam A. (2012). Large excitonic effects in monolayers of molybdenum and tungsten dichalcogenides. Phys. Rev. B.

[26] Jin W., Yeh P.C., Zaki N., Zhang D., Sadowski J.T., Al-Mahboob A., van der Zande A.M., Chenet D.A., Dadap J.I., Herman I.P. (2013). Direct Measurement of the Thickness-Dependent Electronic Band Structure of *MoS*_2_ Using Angle-Resolved Photoemission Spectroscopy. Phys. Rev. Lett..

[27] Coehoorn R., Haas C., de Groot R.A. (1987). Electronic structure of *MoSe*_2_, *MoS*_2_, and *WSe*_2_. II. The nature of the optical band gaps. Phys. Rev. B.

[28] Xiao D., Liu G.B., Feng W., Xu X., Yao W. (2012). Coupled Spin and Valley Physics in Monolayers of *MoS*_2_ and Other Group-VI Dichalcogenides. Phys. Rev. Lett..

[29] Cao T., Wang G., Han W., Ye H., Zhu C., Shi J., Niu Q., Tan P., Wang E., Liu B. (2012). Valley-selective circular dichroism of monolayer molybdenum disulphide. Nat. Commun..

[30] Mak K.F., He K., Shan J., Heinz T.F. (2012). Control of valley polarization in monolayer MoS_2_ by optical helicity. Nat. Nanotechnol..

[31] Sallen G., Bouet L., Marie X., Wang G., Zhu C.R., Han W.P., Lu Y., Tan P.H., Amand T., Liu B.L. (2012). Robust optical emission polarization in MoS_2_ monolayers through selective valley excitation. Phys. Rev. B.

[32] Zeng H., Dai J., Yao W., Xiao D., Cui X. (2012). Valley polarization in MoS_2_ monolayers by optical pumping. Nat. Nanotechnol..

[33] Wang G., Palleau E., Amand T., Tongay S., Marie X., Urbaszek B. (2015). Polarization and time-resolved photoluminescence spectroscopy of excitons in MoSe_2_ monolayers. Appl. Phys. Lett..

[34] Zhang Y., Oka T., Suzuki R., Ye J., Iwasa Y. (2014). Electrically switchable chiral light-emitting transistor. Science.

[35] Kioseoglou G., Hanbicki A.T., Currie M., Friedman A.L., Gunlycke D., Jonker B.T. (2012). Valley polarization and intervalley scattering in monolayer MoS_2_. Appl. Phys. Lett..

[36] Jones A.M., Yu H., Ghimire N.J., Wu S., Aivazian G., Ross J.S., Zhao B., Yan J., Mandrus D.G., Xiao D. (2013). Optical generation of excitonic valley coherence in monolayer WSe_2_. Nat. Nanotechnol..

[37] Wang G., Marie X., Liu B.L., Amand T., Robert C., Cadiz F., Renucci P., Urbaszek B. (2016). Control of Exciton Valley Coherence in Transition Metal Dichalcogenide Monolayers. Phys. Rev. Lett..

[38] Hao K., Moody G., Wu F., Dass C.K., Xu L., Chen C.H., Sun L., Li M.Y., Li L.J., MacDonald A.H. (2016). Direct measurement of exciton valley coherence in monolayer WSe_2_. Nat. Phys..

[39] Srivastava A., Sidler M., Allain A.V., Lembke D.S., Kis A., Imamoglu A. (2015). Valley Zeeman effect in elementary optical excitations of monolayer WSe_2_. Nat. Phys..

[40] Aivazian G., Gong Z., Jones A.M., Chu R.L., Yan J., Mandrus D.G., Zhang C., Cobden D., Yao W., Xu X. (2015). Magnetic control of valley pseudospin in monolayer WSe_2_. Nat. Phys..

[41] Scrace T., Tsai Y., Barman B., Schweidenback L., Petrou A., Kioseoglou G., Ozfidan I., Korkusiński M., Hawrylak P. (2015). Magnetoluminescence and valley polarized state of a two-dimensional electron gas in WS_2_ monolayers. Nat. Nanotechnol..

[42] Braz J.E.H., Amorim B., Castro E.V. (2018). Valley-polarized magnetic state in hole-doped monolayers of transition-metal dichalcogenides. Phys. Rev. B.

[43] Roch J.G., Froehlicher G., Leisgang N., Makk P., Watanabe K., Taniguchi T., Warburton R.J. (2019). Spin-polarized electrons in monolayer MoS_2_. Nat. Nanotechnol..

[44] Miserev D., Klinovaja J., Loss D. (2019). Exchange intervalley scattering and magnetic phase diagram of transition metal dichalcogenide monolayers. Phys. Rev. B.

[45] He K., Kumar N., Zhao L., Wang Z., Mak K.F., Zhao H., Shan J. (2014). Tightly Bound Excitons in Monolayer *WSe*_2_. Phys. Rev. Lett..

[46] Arora A., Koperski M., Nogajewski K., Marcus J., Faugeras C., Potemski M. (2015). Excitonic resonances in thin films of WSe_2_: From monolayer to bulk material. Nanoscale.

[47] Chernikov A., Berkelbach T.C., Hill H.M., Rigosi A., Li Y., Aslan O.B., Reichman D.R., Hybertsen M.S., Heinz T.F. (2014). Exciton Binding Energy and Nonhydrogenic Rydberg Series in Monolayer *WS*_2_. Phys. Rev. Lett..

[48] Hill H.M., Rigosi A.F., Roquelet C., Chernikov A., Berkelbach T.C., Reichman D.R., Hybertsen M.S., Brus L.E., Heinz T.F. (2015). Observation of Excitonic Rydberg States in Monolayer MoS_2_ and WS_2_ by Photoluminescence Excitation Spectroscopy. Nano Lett..

[49] Arora A., Nogajewski K., Molas M., Koperski M., Potemski M. (2015). Exciton band structure in layered MoSe_2_: From a monolayer to the bulk limit. Nanoscale.

[50] Wang G., Marie X., Gerber I., Amand T., Lagarde D., Bouet L., Vidal M., Balocchi A., Urbaszek B. (2015). Giant Enhancement of the Optical Second-Harmonic Emission of *WSe*_2_ Monolayers by Laser Excitation at Exciton Resonances. Phys. Rev. Lett..

[51] Stier A.V., McCreary K.M., Jonker B.T., Kono J., Crooker S.A. (2016). Exciton diamagnetic shifts and valley Zeeman effects in monolayer WS_2_ and MoS_2_ to 65 Tesla. Nat. Commun..

[52] Stier A.V., Wilson N.P., Velizhanin K.A., Kono J., Xu X., Crooker S.A. (2018). Magnetooptics of Exciton Rydberg States in a Monolayer Semiconductor. Phys. Rev. Lett..

[53] Molas M.R., Slobodeniuk A.O., Nogajewski K., Bartos M., Bala L., Babiński A., Watanabe K., Taniguchi T., Faugeras C., Potemski M. (2019). Energy Spectrum of Two-Dimensional Excitons in a Nonuniform Dielectric Medium. Phys. Rev. Lett..

[54] Goryca M., Li J., Stier A., Crooker S., Taniguchi T., Watanabe K., Courtade E., Shree S., Robert C., Urbaszek B. (2019). Revealing exciton masses and dielectric properties of monolayer semiconductors with high magnetic fields. Nat. Commun..

[55] Delhomme A., Butseraen G., Zheng B., Marty L., Bouchiat V., Molas M.R., Pan A., Watanabe K., Taniguchi T., Ouerghi A. (2019). Magneto-spectroscopy of exciton Rydberg states in a CVD grown WSe_2_ monolayer. Appl. Phys. Lett..

[56] Bieniek M., Korkusiński M., Szulakowska L., Potasz P., Ozfidan I., Hawrylak P. (2018). Band nesting, massive Dirac fermions, and valley Landé and Zeeman effects in transition metal dichalcogenides: A tight-binding model. Phys. Rev. B.

[57] Zhang X.X., You Y., Zhao S.Y.F., Heinz T.F. (2015). Experimental Evidence for Dark Excitons in Monolayer *WSe*_2_. Phys. Rev. Lett..

[58] Wang G., Robert C., Suslu A., Chen B., Yang S., Alamdari S., Gerber I.C., Amand T., Marie X., Tongay S. (2015). Spin-orbit engineering in transition metal dichalcogenide alloy monolayers. Nat. Commun..

[59] Wang G., Robert C., Glazov M.M., Cadiz F., Courtade E., Amand T., Lagarde D., Taniguchi T., Watanabe K., Urbaszek B. (2017). In-Plane Propagation of Light in Transition Metal Dichalcogenide Monolayers: Optical Selection Rules. Phys. Rev. Lett..

[60] Robert C., Amand T., Cadiz F., Lagarde D., Courtade E., Manca M., Taniguchi T., Watanabe K., Urbaszek B., Marie X. (2017). Fine structure and lifetime of dark excitons in transition metal dichalcogenide monolayers. Phys. Rev. B.

[61] Tang Y., Mak K.F., Shan J. (2019). Long valley lifetime of dark excitons in single-layer WSe_2_. Nat. Commun..

[62] Park K.D., Jiang T., Clark G., Xu X., Raschke M.B. (2018). Radiative control of dark excitons at room temperature by nano-optical antenna-tip Purcell effect. Nat. Nanotechnol..

[63] Zhou Y., Scuri G., Wild D.S., High A.A., Dibos A., Jauregui L.A., Shu C., De Greve K., Pistunova K., Joe A.Y. (2017). Probing dark excitons in atomically thin semiconductors via near-field coupling to surface plasmon polaritons. Nat. Nanotechnol..

[64] Molas M.R., Slobodeniuk A.O., Kazimierczuk T., Nogajewski K., Bartos M., Kapuściński P., Oreszczuk K., Watanabe K., Taniguchi T., Faugeras C. (2019). Probing and Manipulating Valley Coherence of Dark Excitons in Monolayer *WSe*_2_. Phys. Rev. Lett..

[65] Withers F., Del Pozo-Zamudio O., Schwarz S., Dufferwiel S., Walker P.M., Godde T., Rooney A.P., Gholinia A., Woods C.R., Blake P. (2015). WSe_2_ Light-Emitting Tunneling Transistors with Enhanced Brightness at Room Temperature. Nano Lett..

[66] Zhang X.X., Cao T., Lu Z., Lin Y.C., Zhang F., Wang Y., Li Z., Hone J.C., Robinson J.A., Smirnov D. (2017). Magnetic brightening and control of dark excitons in monolayer WSe_2_. Nat. Nanotechnol..

[67] Molas M.R., Faugeras C., Slobodeniuk A.O., Nogajewski K., Bartos M., Basko D.M., Potemski M. (2017). Brightening of dark excitons in monolayers of semiconducting transition metal dichalcogenides. 2D Mater..

[68] Lu Z., Rhodes D., Li Z., Tuan D.V., Jiang Y., Ludwig J., Jiang Z., Lian Z., Shi S.F., Hone J. (2019). Magnetic field mixing and splitting of bright and dark excitons in monolayer MoSe_2_. 2D Mater..

[69] Li Z., Wang T., Jin C., Lu Z., Lian Z., Meng Y., Blei M., Gao S., Taniguchi T., Watanabe K. (2019). Emerging photoluminescence from the dark-exciton phonon replica in monolayer WSe_2_. Nat. Commun..

[70] Ye Z., Cao T., O’Brien K., Zhu H., Yin X., Wang Y., Louie S.G., Zhang X. (2014). Probing excitonic dark states in single-layer tungsten disulphide. Nature.

[71] Berghauser G., Knorr A., Malic E. (2017). Optical fingerprint of dark 2p-states in transition metal dichalcogenides. 2D Mater..

[72] Pöllmann C., Steinleitner P., Leierseder U., Nagler P., Plechinger G., Porer M., Bratschitsch R., Schüller C., Korn T., Huber R. (2015). Resonant internal quantum transitions and femtosecond radiative decay of excitons in monolayer WSe_2_. Nat. Mater..

[73] Cha S., Sung J.H., Sim S., Park J., Heo H., Jo M.H., Choi H. (2016). 1s-intraexcitonic dynamics in monolayer MoS_2_ probed by ultrafast mid-infrared spectroscopy. Nat. Commun..

[74] Steinleitner P., Merkl P., Graf A., Nagler P., Watanabe K., Taniguchi T., Zipfel J., Schüller C., Korn T., Chernikov A. (2018). Dielectric Engineering of Electronic Correlations in a van der Waals Heterostructure. Nano Lett..

[75] Berghäuser G., Steinleitner P., Merkl P., Huber R., Knorr A., Malic E. (2018). Mapping of the dark exciton landscape in transition metal dichalcogenides. Phys. Rev. B.

[76] Brem S., Zipfel J., Selig M., Raja A., Waldecker L., Ziegler J.D., Taniguchi T., Watanabe K., Chernikov A., Malic E. (2019). Intrinsic lifetime of higher excitonic states in tungsten diselenide monolayers. Nanoscale.

[77] Merkl P., Mooshammer F., Steinleitner P., Girnghuber A., Lin K.Q., Nagler P., Holler J., Schüller C., Lupton J.M., Korn T. (2019). Ultrafast transition between exciton phases in van der Waals heterostructures. Nat. Mater..

[78] Yong C.K., Utama M.I.B., Ong C.S., Cao T., Regan E.C., Horng J., Shen Y., Cai H., Watanabe K., Taniguchi T. (2019). Valley-dependent exciton fine structure and Autler–Townes doublets from Berry phases in monolayer MoSe_2_. Nat. Mater..

[79] Berkelbach T.C., Hybertsen M.S., Reichman D.R. (2015). Bright and dark singlet excitons via linear and two-photon spectroscopy in monolayer transition-metal dichalcogenides. Phys. Rev. B.

[80] Feierabend M., Berghäuser G., Knorr A., Malic E. (2017). Proposal for dark exciton based chemical sensors. Nat. Commun..

[81] Christiansen D., Selig M., Berghäuser G., Schmidt R., Niehues I., Schneider R., Arora A., de Vasconcellos S.M., Bratschitsch R., Malic E. (2017). Phonon Sidebands in Monolayer Transition Metal Dichalcogenides. Phys. Rev. Lett..

[82] Selig M., Berghäuser G., Raja A., Nagler P., Schüller C., Heinz T.F., Korn T., Chernikov A., Malic E., Knorr A. (2016). Excitonic linewidth and coherence lifetime in monolayer transition metal dichalcogenides. Nat. Commun..

[83] Peng G.H., Lo P.Y., Li W.H., Huang Y.C., Chen Y.H., Lee C.H., Yang C.K., Cheng S.J. (2019). Distinctive Signatures of the Spin- and Momentum-Forbidden Dark Exciton States in the Photoluminescence of Strained WSe_2_ Monolayers under Thermalization. Nano Lett..

[84] Lampert M.A. (1958). Mobile and Immobile Effective-Mass-Particle Complexes in Nonmetallic Solids. Phys. Rev. Lett..

[85] Kheng K., Cox R.T., d’ Aubigné M.Y., Bassani F., Saminadayar K., Tatarenko S. (1993). Observation of negatively charged excitons *X*^−^ in semiconductor quantum wells. Phys. Rev. Lett..

[86] Hawrylak P. (1991). Optical properties of a two-dimensional electron gas: Evolution of spectra from excitons to Fermi-edge singularities. Phys. Rev. B.

[87] Narvaez G.A., Hawrylak P., Brum J.A. (2001). The role of finite hole mass in the negatively charged exciton in two dimensions. Phys. E Low-Dimens. Syst. Nanostruct..

[88] Mak K.F., He K., Lee C., Lee G.H., Hone J., Heinz T.F., Shan J. (2013). Tightly bound trions in monolayer MoS_2_. Nat. Mater..

[89] Mitioglu A.A., Plochocka P., Jadczak J.N., Escoffier W., Rikken G.L.J.A., Kulyuk L., Maude D.K. (2013). Optical manipulation of the exciton charge state in single-layer tungsten disulfide. Phys. Rev. B.

[90] Lui C.H., Frenzel A.J., Pilon D.V., Lee Y.H., Ling X., Akselrod G.M., Kong J., Gedik N. (2014). Trion-Induced Negative Photoconductivity in Monolayer *MoS*_2_. Phys. Rev. Lett..

[91] Singh A., Moody G., Tran K., Scott M.E., Overbeck V., Berghäuser G., Schaibley J., Seifert E.J., Pleskot D., Gabor N.M. (2016). Trion formation dynamics in monolayer transition metal dichalcogenides. Phys. Rev. B.

[92] Zhang C., Wang H., Chan W., Manolatou C., Rana F. (2014). Absorption of light by excitons and trions in monolayers of metal dichalcogenide *Mo*S_2_: Experiments and theory. Phys. Rev. B.

[93] Rezk A.R., Carey B., Chrimes A.F., Lau D.W.M., Gibson B.C., Zheng C., Fuhrer M.S., Yeo L.Y., Kalantar-zadeh K. (2016). Acoustically-Driven Trion and Exciton Modulation in Piezoelectric Two-Dimensional MoS_2_. Nano Lett..

[94] Plechinger G., Nagler P., Kraus J., Paradiso N., Strunk C., Schüller C., Korn T. (2015). Identification of excitons, trions and biexcitons in single-layer WS_2_. Phys. Status Solidi (RRL)—Rapid Res. Lett..

[95] Cuadra J., Baranov D.G., Wersall M., Verre R., Antosiewicz T.J., Shegai T. (2018). Observation of Tunable Charged Exciton Polaritons in Hybrid Monolayer WS_2_ Plasmonic Nanoantenna System. Nano Lett..

[96] Jadczak J., Delgado A., Bryja L., Huang Y.S., Hawrylak P. (2017). Robust high-temperature trion emission in monolayers of *Mo*(S_*y*_Se_1−*y*_)_2_ alloys. Phys. Rev. B.

[97] Jadczak J., Kutrowska-Girzycka J., Kapuściński P., Huang Y.S., Wójs A., Bryja L. (2017). Probing of free and localized excitons and trions in atomically thin WSe_2_, WS_2_, MoSe_2_ and MoS_2_ in photoluminescence and reflectivity experiments. Nanotechnology.

[98] Nan H., Wang Z., Wang W., Liang Z., Lu Y., Chen Q., He D., Tan P., Miao F., Wang X. (2014). Strong Photoluminescence Enhancement of MoS_2_ through Defect Engineering and Oxygen Bonding. ACS Nano.

[99] Mouri S., Miyauchi Y., Matsuda K. (2013). Tunable Photoluminescence of Monolayer MoS_2_ via Chemical Doping. Nano Lett..

[100] Scheuschner N., Ochedowski O., Kaulitz A.M., Gillen R., Schleberger M., Maultzsch J. (2014). Photoluminescence of freestanding single- and few-layer MoS_2_. Phys. Rev. B.

[101] Godde T., Schmidt D., Schmutzler J., Aßmann M., Debus J., Withers F., Alexeev E.M., Del Pozo-Zamudio O., Skrypka O.V., Novoselov K.S. (2016). Exciton and trion dynamics in atomically thin *MoSe*_2_ and *WSe*_2_: Effect of localization. Phys. Rev. B.

[102] Ross J.S., Wu S., Yu H., Ghimire N.J., Jones A.M., Aivazian G., Yan J., Mandrus D.G., Xiao D., Yao W. (2013). Electrical control of neutral and charged excitons in a monolayer semiconductor. Nat. Commun..

[103] Ross J.S., Klement P., Jones A.M., Ghimire N.J., Yan J., Mandrus D.G., Taniguchi T., Watanabe K., Kitamura K., Yao W. (2014). Electrically tunable excitonic light-emitting diodes based on monolayer WSe_2_ p-n junctions. Nat. Nanotechnol..

[104] Shang J., Shen X., Cong C., Peimyoo N., Cao B., Eginligil M., Yu T. (2015). Observation of Excitonic Fine Structure in a 2D Transition-Metal Dichalcogenide Semiconductor. ACS Nano.

[105] Yao K., Yan A., Kahn S., Suslu A., Liang Y., Barnard E.S., Tongay S., Zettl A., Borys N.J., Schuck P.J. (2017). Optically Discriminating Carrier-Induced Quasiparticle Band Gap and Exciton Energy Renormalization in Monolayer *MoS*_2_. Phys. Rev. Lett..

[106] Yang J., Lü T., Myint Y.W., Pei J., Macdonald D., Zheng J.C., Lu Y. (2015). Robust Excitons and Trions in Monolayer MoTe_2_. ACS Nano.

[107] Courtade E., Semina M., Manca M., Glazov M.M., Robert C., Cadiz F., Wang G., Taniguchi T., Watanabe K., Pierre M. (2017). Charged excitons in monolayer *WSe*_2_: Experiment and theory. Phys. Rev. B.

[108] Liu E., van Baren J., Lu Z., Altaiary M.M., Taniguchi T., Watanabe K., Smirnov D., Lui C.H. (2019). Gate Tunable Dark Trions in Monolayer *WSe*_2_. Phys. Rev. Lett..

[109] Back P., Sidler M., Cotlet O., Srivastava A., Takemura N., Kroner M., Imamoğlu A. (2017). Giant Paramagnetism-Induced Valley Polarization of Electrons in Charge-Tunable Monolayer *MoSe*_2_. Phys. Rev. Lett..

[110] Plechinger G., Nagler P., Arora A., Schmidt R., Chernikov A., del Águila A.G., Christianen P.C.M., Bratschitsch R., Schüller C., Korn T. (2016). Trion fine structure and coupled spin-valley dynamics in monolayer tungsten disulfide. Nat. Commun..

[111] Vaclavkova D., Wyzula J., Nogajewski K., Bartos M., Slobodeniuk A.O., Faugeras C., Potemski M., Molas M.R. (2018). Singlet and triplet trions in WS_2_ monolayer encapsulated in hexagonal boron nitride. Nanotechnology.

[112] Boulesbaa A., Huang B., Wang K., Lin M.W., Mahjouri-Samani M., Rouleau C., Xiao K., Yoon M., Sumpter B., Puretzky A. (2015). Observation of two distinct negative trions in tungsten disulfide monolayers. Phys. Rev. B.

[113] Orsi Gordo V., Balanta M.A.G., Galvão Gobato Y., Covre F.S., Galeti H.V.A., Iikawa F., Couto O.D.D., Qu F., Henini M., Hewak D.W. (2018). Revealing the nature of low-temperature photoluminescence peaks by laser treatment in van der Waals epitaxially grown WS_2_ monolayers. Nanoscale.

[114] Molas M.R., Nogajewski K., Slobodeniuk A.O., Binder J., Bartos M., Potemski M. (2017). The optical response of monolayer, few-layer and bulk tungsten disulfide. Nanoscale.

[115] Li Z., Wang T., Lu Z., Khatoniar M., Lian Z., Meng Y., Blei M., Taniguchi T., Watanabe K., McGill S.A. (2019). Direct Observation of Gate-Tunable Dark Trions in Monolayer WSe_2_. Nano Lett..

[116] Jadczak J., Kutrowska-Girzycka J., Bieniek M., Kazimierczuk T., Kossacki P., Schindler J., Debus J., Watanabe K., Taniguchi T., Ho C. (2021). Probing negatively charged and neutral excitons in MoS_2_/hBN and hBN/MoS_2_/hBN van der Waals heterostructures. Nanotechnology.

[117] Arora A., Deilmann T., Reichenauer T., Kern J., Michaelis de Vasconcellos S., Rohlfing M., Bratschitsch R. (2019). Excited-State Trions in Monolayer *WS*_2_. Phys. Rev. Lett..

[118] Jones A.M., Yu H., Schaibley J.R., Yan J., Mandrus D.G., Taniguchi T., Watanabe K., Dery H., Yao W., Xu X. (2016). Excitonic luminescence upconversion in a two-dimensional semiconductor. Nat. Phys..

[119] Jadczak J., Bryja L., Kutrowska-Girzycka J., Kapuscinski P., Bieniek M., Huang Y.S., Hawrylak P. (2019). Room temperature multi-phonon upconversion photoluminescence in monolayer semiconductor WS_2_. Nat. Commun..

[120] Mai C., Barrette A., Yu Y., Semenov Y.G., Kim K.W., Cao L., Gundogdu K. (2014). Many-Body Effects in Valleytronics: Direct Measurement of Valley Lifetimes in Single-Layer MoS_2_. Nano Lett..

[121] You Y., Zhang X.X., Berkelbach T.C., Hybertsen M.S., Reichman D.R., Heinz T.F. (2015). Observation of biexcitons in monolayer WSe_2_. Nat. Phys..

[122] Sie E.J., Frenzel A.J., Lee Y.H., Kong J., Gedik N. (2015). Intervalley biexcitons and many-body effects in monolayer *MoS*_2_. Phys. Rev. B.

[123] Lee H.S., Kim M.S., Kim H., Lee Y.H. (2016). Identifying multiexcitons in *Mo*S_2_ monolayers at room temperature. Phys. Rev. B.

[124] Okada M., Miyauchi Y., Matsuda K., Taniguchi T., Watanabe K., Shinohara H., Kitaura R. (2017). Observation of biexcitonic emission at extremely low power density in tungsten disulfide atomic layers grown on hexagonal boron nitride. Sci. Rep..

[125] Nagler P., Ballottin M.V., Mitioglu A.A., Durnev M.V., Taniguchi T., Watanabe K., Chernikov A., Schüller C., Glazov M.M., Christianen P.C.M. (2018). Zeeman Splitting and Inverted Polarization of Biexciton Emission in Monolayer *WS*_2_. Phys. Rev. Lett..

[126] Paradisanos I., Germanis S., Pelekanos N.T., Fotakis C., Kymakis E., Kioseoglou G., Stratakis E. (2017). Room temperature observation of biexcitons in exfoliated WS_2_ monolayers. Appl. Phys. Lett..

[127] Pei J., Yang J., Wang X., Wang F., Mokkapati S., Lü T., Zheng J.C., Qin Q., Neshev D., Tan H.H. (2017). Excited State Biexcitons in Atomically Thin MoSe_2_. ACS Nano.

[128] Li Z., Wang T., Lu Z., Jin C., Chen Y., Meng Y., Lian Z., Taniguchi T., Watanabe K., Zhang S. (2018). Revealing the biexciton and trion-exciton complexes in BN encapsulated WSe_2_. Nat. Commun..

[129] Barbone M., Montblanch A.R.P., Kara D.M., Palacios-Berraquero C., Cadore A.R., De Fazio D., Pingault B., Mostaani E., Li H., Chen B. (2018). Charge-tuneable biexciton complexes in monolayer WSe_2_. Nat. Commun..

[130] Paur M., Molina-Mendoza A.J., Bratschitsch R., Watanabe K., Taniguchi T., Mueller T. (2019). Electroluminescence from multi-particle exciton complexes in transition metal dichalcogenide semiconductors. Nat. Commun..

[131] Chen S.Y., Goldstein T., Taniguchi T., Watanabe K., Yan J. (2018). Coulomb-bound four- and five-particle intervalley states in an atomically-thin semiconductor. Nat. Commun..

[132] Hao K., Specht J.F., Nagler P., Xu L., Tran K., Singh A., Dass C.K., Schüller C., Korn T., Richter M. (2017). Neutral and charged inter-valley biexcitons in monolayer MoSe_2_. Nat. Commun..

[133] Liu W., Lee B., Naylor C.H., Ee H.S., Park J., Johnson A.T.C., Agarwal R. (2016). Strong Exciton–Plasmon Coupling in MoS_2_ Coupled with Plasmonic Lattice. Nano Lett..

[134] Van Tuan D., Scharf B., Žutić I., Dery H. (2017). Marrying Excitons and Plasmons in Monolayer Transition-Metal Dichalcogenides. Phys. Rev. X.

[135] Sidler M., Back P., Cotlet O., Srivastava A., Fink T., Kroner M., Demler E., Imamoglu A. (2017). Fermi polaron-polaritons in charge-tunable atomically thin semiconductors. Nat. Phys..

[136] Geim A.K., Grigorieva I.V. (2013). Van der Waals heterostructures. Nature.

[137] Ponomarenko L.A., Geim A.K., Zhukov A.A., Jalil R., Morozov S.V., Novoselov K.S., Grigorieva I.V., Hill E.H., Cheianov V.V., Fal’ko V.I. (2011). Tunable metal-insulator transition in double-layer graphene heterostructures. Nat. Phys..

[138] Georgiou T., Jalil R., Belle B.D., Britnell L., Gorbachev R.V., Morozov S.V., Kim Y.J., Gholinia A., Haigh S.J., Makarovsky O. (2013). Vertical field-effect transistor based on graphene-WS_2_ heterostructures for flexible and transparent electronics. Nat. Nanotechnol..

[139] Robert C., Semina M.A., Cadiz F., Manca M., Courtade E., Taniguchi T., Watanabe K., Cai H., Tongay S., Lassagne B. (2018). Optical spectroscopy of excited exciton states in *MoS*_2_ monolayers in van der Waals heterostructures. Phys. Rev. Mater..

[140] Jin C., Ma E.Y., Karni O., Regan E.C., Wang F., Heinz T.F. (2018). Ultrafast dynamics in van der Waals heterostructures. Nat. Nanotechnol..

[141] Fang H., Battaglia C., Carraro C., Nemsak S., Ozdol B., Kang J.S., Bechtel H.A., Desai S.B., Kronast F., Unal A.A. (2014). Strong interlayer coupling in van der Waals heterostructures built from single-layer chalcogenides. Proc. Natl. Acad. Sci. USA.

[142] Rivera P., Schaibley J.R., Jones A.M., Ross J.S., Wu S., Aivazian G., Klement P., Seyler K., Clark G., Ghimire N.J. (2015). Observation of long-lived interlayer excitons in monolayer MoSe_2_-WSe_2_ heterostructures. Nat. Commun..

[143] Chen H., Wen X., Zhang J., Wu T., Gong Y., Zhang X., Yuan J., Yi C., Lou J., Ajayan P.M. (2016). Ultrafast formation of interlayer hot excitons in atomically thin MoS_2_/WS_2_ heterostructures. Nat. Commun..

[144] Baranowski M., Surrente A., Klopotowski L., Urban J.M., Zhang N., Maude D.K., Wiwatowski K., Mackowski S., Kung Y.C., Dumcenco D. (2017). Probing the Interlayer Exciton Physics in a MoS_2_/MoSe_2_/MoS_2_ van der Waals Heterostructure. Nano Lett..

[145] Miller B., Steinhoff A., Pano B., Klein J., Jahnke F., Holleitner A., Wurstbauer U. (2017). Long-Lived Direct and Indirect Interlayer Excitons in van der Waals Heterostructures. Nano Lett..

[146] Nagler P., Plechinger G., Ballottin M.V., Mitioglu A., Meier S., Paradiso N., Strunk C., Chernikov A., Christianen P.C.M., Schüller C. (2017). Interlayer exciton dynamics in a dichalcogenide monolayer heterostructure. 2D Mater..

[147] Wang T., Miao S., Li Z., Meng Y., Lu Z., Lian Z., Blei M., Taniguchi T., Watanabe K., Tongay S. (2020). Giant Valley-Zeeman Splitting from Spin-Singlet and Spin-Triplet Interlayer Excitons in WSe_2_/MoSe_2_ Heterostructure. Nano Lett..

[148] Choi J., Florian M., Steinhoff A., Erben D., Tran K., Kim D.S., Sun L., Quan J., Claassen R., Majumder S. (2021). Twist Angle-Dependent Interlayer Exciton Lifetimes in van der Waals Heterostructures. Phys. Rev. Lett..

[149] Nagler P., Ballottin M.V., Mitioglu A.A., Mooshammer F., Paradiso N., Strunk C., Huber R., Chernikov A., Christianen P.C., Schüller C. (2017). Giant magnetic splitting inducing near-unity valley polarization in van der Waals heterostructures. Nat. Commun..

[150] Hsu W.T., Lu L.S., Wu P.H., Lee M.H., Chen P.J., Wu P.Y., Chou Y.C., Jeng H.T., Li L.J., Chu M.W. (2018). Negative circular polarization emissions from WSe_2_/MoSe_2_ commensurate heterobilayers. Nat. Commun..

[151] Hanbicki A.T., Chuang H.J., Rosenberger M.R., Hellberg C.S., Sivaram S.V., McCreary K.M., Mazin I.I., Jonker B.T. (2018). Double indirect interlayer exciton in a MoSe_2_/WSe_2_ van der Waals heterostructure. Acs Nano.

[152] Wang J., Ardelean J., Bai Y., Steinhoff A., Florian M., Jahnke F., Xu X., Kira M., Hone J., Zhu X.Y. (2019). Optical generation of high carrier densities in 2D semiconductor heterobilayers. Sci. Adv..

[153] Ciarrocchi A., Unuchek D., Avsar A., Watanabe K., Taniguchi T., Kis A. (2019). Polarization switching and electrical control of interlayer excitons in two-dimensional van der Waals heterostructures. Nat. Photonics.

[154] Zhang L., Gogna R., Burg G.W., Horng J., Paik E., Chou Y.H., Kim K., Tutuc E., Deng H. (2019). Highly valley-polarized singlet and triplet interlayer excitons in van der Waals heterostructure. Phys. Rev. B.

[155] Li W., Lu X., Dubey S., Devenica L., Srivastava A. (2020). Dipolar interactions between localized interlayer excitons in van der Waals heterostructures. Nat. Mater..

[156] Joe A.Y., Jauregui L.A., Pistunova K., Mier Valdivia A.M., Lu Z., Wild D.S., Scuri G., De Greve K., Gelly R.J., Zhou Y. (2021). Electrically controlled emission from singlet and triplet exciton species in atomically thin light-emitting diodes. Phys. Rev. B.

[157] Sigl L., Sigger F., Kronowetter F., Kiemle J., Klein J., Watanabe K., Taniguchi T., Finley J.J., Wurstbauer U., Holleitner A.W. (2020). Signatures of a degenerate many-body state of interlayer excitons in a van der Waals heterostack. Phys. Rev. Res..

[158] Rivera P., Seyler K.L., Yu H., Schaibley J.R., Yan J., Mandrus D.G., Yao W., Xu X. (2016). Valley-polarized exciton dynamics in a 2D semiconductor heterostructure. Science.

[159] Lorchat E., Selig M., Katsch F., Yumigeta K., Tongay S., Knorr A., Schneider C., Höfling S. (2021). Excitons in Bilayer MoS_2_ Displaying a Colossal Electric Field Splitting and Tunable Magnetic Response. Phys. Rev. Lett..

[160] Jauregui L.A., Joe A.Y., Pistunova K., Wild D.S., High A.A., Zhou Y., Scuri G., De Greve K., Sushko A., Yu C.H. (2019). Electrical control of interlayer exciton dynamics in atomically thin heterostructures. Science.

[161] Calman E.V., Fowler-Gerace L.H., Choksy D.J., Butov L.V., Nikonov D.E., Young I.A., Hu S., Mishchenko A., Geim A.K. (2020). Indirect Excitons and Trions in MoSe_2_/WSe_2_ van der Waals Heterostructures. Nano Lett..

[162] Rivera P., Yu H., Seyler K.L., Wilson N.P., Yao W., Xu X. (2018). Interlayer valley excitons in heterobilayers of transition metal dichalcogenides. Nat. Nanotechnol..

[163] Zhu X., He J., Zhang R., Cong C., Zheng Y., Zhang H., Zhang S., Chen L. (2020). Effects of dielectric screening on the excitonic and critical points properties of WS_2_/MoS_2_ heterostructures. Nanoscale.

[164] Fogler M.M., Butov L.V., Novoselov K.S. (2014). High-temperature superfluidity with indirect excitons in van der Waals heterostructures. Nat. Commun..

[165] Calman E.V., Fogler M.M., Butov L.V., Hu S., Mishchenko A., Geim A.K. (2018). Indirect excitons in van der Waals heterostructures at room temperature. Nat. Commun..

[166] Mouri S., Zhang W., Kozawa D., Miyauchi Y., Eda G., Matsuda K. (2017). Thermal dissociation of inter-layer excitons in MoS_2_/MoSe_2_ hetero-bilayers. Nanoscale.

[167] Deilmann T., Thygesen K.S. (2018). Interlayer Trions in the MoS_2_/WS_2_ van der Waals Heterostructure. Nano Lett..

[168] Vialla F., Danovich M., Ruiz-Tijerina D.A., Massicotte M., Schmidt P., Taniguchi T., Watanabe K., Hunt R.J., Szyniszewski M., Drummond N.D. (2019). Tuning of impurity-bound interlayer complexes in a van der Waals heterobilayer. 2D Mater..

[169] Baek H., Brotons-Gisbert M., Campbell A., Vitale V., Lischner J., Watanabe K., Taniguchi T., Gerardot B.D. (2021). Optical read-out of Coulomb staircases in a moiré superlattice via trapped interlayer trions. Nat. Nanotechnol..

[170] Wang Z., Rhodes D.A., Watanabe K., Taniguchi T., Hone J.C., Shan J., Mak K.F. (2019). Evidence of high-temperature exciton condensation in two-dimensional atomic double layers. Nature.

[171] Van der Zande A.M., Kunstmann J., Chernikov A., Chenet D.A., You Y., Zhang X., Huang P.Y., Berkelbach T.C., Wang L., Zhang F. (2014). Tailoring the Electronic Structure in Bilayer Molybdenum Disulfide via Interlayer Twist. Nano Lett..

[172] Liu K., Zhang L., Cao T., Jin C., Qiu D., Zhou Q., Zettl A., Yang P., Louie S.G., Wang F. (2014). Evolution of interlayer coupling in twisted molybdenum disulfide bilayers. Nat. Commun..

[173] Huang S., Ling X., Liang L., Kong J., Terrones H., Meunier V., Dresselhaus M.S. (2014). Probing the Interlayer Coupling of Twisted Bilayer MoS_2_ Using Photoluminescence Spectroscopy. Nano Lett..

[174] Yeh P.C., Jin W., Zaki N., Kunstmann J., Chenet D., Arefe G., Sadowski J.T., Dadap J.I., Sutter P., Hone J. (2016). Direct Measurement of the Tunable Electronic Structure of Bilayer MoS_2_ by Interlayer Twist. Nano Lett..

[175] Huang S., Liang L., Ling X., Puretzky A.A., Geohegan D.B., Sumpter B.G., Kong J., Meunier V., Dresselhaus M.S. (2016). Low-Frequency Interlayer Raman Modes to Probe Interface of Twisted Bilayer MoS_2_. Nano Lett..

[176] Liao M., Wei Z., Du L., Wang Q., Tang J., Yu H., Wu F., Zhao J., Xu X., Han B. (2020). Precise control of the interlayer twist angle in large scale MoS_2_ homostructures. Nat. Commun..

[177] Grzeszczyk M., Szpakowski J., Slobodeniuk A., Kazimierczuk T., Bhatnagar M., Taniguchi T., Watanabe K., Kossacki P., Potemski M., Babiński A. (2021). The optical response of artificially twisted MoS_2_ bilayers. Sci. Rep..

[178] Tran K., Choi J., Singh A. (2020). Moiré and beyond in transition metal dichalcogenide twisted bilayers. 2D Mater..

[179] Brotons-Gisbert M., Baek H., Campbell A., Watanabe K., Taniguchi T., Gerardot B. (2021). Moire-Trapped Interlayer Trions in a Charge-Tunable WSe_2_/MoSe_2_ Heterobilayer. Phys. Rev. X.

[180] Ruiz-Tijerina D.A., Fal’ko V.I. (2019). Interlayer hybridization and moiré superlattice minibands for electrons and excitons in heterobilayers of transition-metal dichalcogenides. Phys. Rev. B.

[181] Kunstmann J., Mooshammer F., Nagler P., Chaves A., Stein F., Paradiso N., Plechinger G., Strunk C., Schüller C., Seifert G. (2018). Momentum-space indirect interlayer excitons in transition-metal dichalcogenide van der Waals heterostructures. Nat. Phys..

[182] Tran K., Moody G., Wu F., Lu X., Choi J., Kim K., Rai A., Sanchez D.A., Quan J., Singh A. (2019). Evidence for moiré excitons in van der Waals heterostructures. Nature.

[183] Seyler K.L., Rivera P., Yu H., Wilson N.P., Ray E.L., Mandrus D.G., Yan J., Yao W., Xu X. (2019). Signatures of moiré-trapped valley excitons in MoSe_2_/WSe_2_ heterobilayers. Nature.

[184] Jin C., Regan E.C., Yan A., Iqbal Bakti Utama M., Wang D., Zhao S., Qin Y., Yang S., Zheng Z., Shi S. (2019). Observation of moiré excitons in WSe_2_/WS_2_ heterostructure superlattices. Nature.

[185] Alexeev E.M., Ruiz-Tijerina D.A., Danovich M., Hamer M.J., Terry D.J., Nayak P.K., Ahn S., Pak S., Lee J., Sohn J.I. (2019). Resonantly hybridized excitons in moiré superlattices in van der Waals heterostructures. Nature.

[186] Choi J., Hsu W.T., Lu L.S., Sun L., Cheng H.Y., Lee M.H., Quan J., Tran K., Wang C.Y., Staab M. (2020). Moiré potential impedes interlayer exciton diffusion in van der Waals heterostructures. Sci. Adv..

[187] Tang Y., Li L., Li T., Xu Y., Liu S., Barmak K., Watanabe K., Taniguchi T., MacDonald A.H., Shan J. (2020). Simulation of Hubbard model physics in WSe_2_/WS_2_ moiré superlattices. Nature.

[188] Regan E.C., Wang D., Jin C., Bakti Utama M.I., Gao B., Wei X., Zhao S., Zhao W., Zhang Z., Yumigeta K. (2020). Mott and generalized Wigner crystal states in WSe_2_/WS_2_ moiré superlattices. Nature.

[189] Zheng Z., Ma Q., Bi Z., de la Barrera S., Liu M.H., Mao N., Zhang Y., Kiper N., Watanabe K., Taniguchi T. (2020). Unconventional ferroelectricity in moiré heterostructures. Nature.

[190] Kennes D.M., Claassen M., Xian L., Georges A., Millis A.J., Hone J., Dean C.R., Basov D.N., Pasupathy A.N., Rubio A. (2021). Moiré heterostructures as a condensed-matter quantum simulator. Nat. Phys..

[191] Jin C., Tao Z., Li T., Xu Y., Tang Y., Zhu J., Liu S., Watanabe K., Taniguchi T., Hone J.C. (2021). Stripe phases in WSe_2_/WS_2_ moiré superlattices. Nat. Mater..

[192] Huang X., Wang T., Miao S., Wang C., Li Z., Lian Z., Taniguchi T., Watanabe K., Okamoto S., Xiao D. (2021). Correlated insulating states at fractional fillings of the WS_2_/WSe_2_ moiré lattice. Nat. Phys..

[193] Salpeter E.E., Bethe H.A. (1951). A Relativistic Equation for Bound-State Problems. Phys. Rev..

[194] Sham L.J., Rice T.M. (1966). Many-Particle Derivation of the Effective-Mass Equation for the Wannier Exciton. Phys. Rev..

[195] Strinati G. (1982). Dynamical Shift and Broadening of Core Excitons in Semiconductors. Phys. Rev. Lett..

[196] Strinati G. (1984). Effects of dynamical screening on resonances at inner-shell thresholds in semiconductors. Phys. Rev. B.

[197] Hanke W., Sham L.J. (1979). Many-Particle Effects in the Optical Excitations of a Semiconductor. Phys. Rev. Lett..

[198] Albrecht S., Onida G., Reining L. (1997). Ab initio calculation of the quasiparticle spectrum and excitonic effects in Li_2_O. Phys. Rev. B.

[199] Albrecht S., Reining L., Del Sole R., Onida G. (1998). Ab Initio Calculation of Excitonic Effects in the Optical Spectra of Semiconductors. Phys. Rev. Lett..

[200] Rohlfing M., Louie S.G. (2000). Electron-hole excitations and optical spectra from first principles. Phys. Rev. B.

[201] Deslippe J., Samsonidze G., Strubbe D.A., Jain M., Cohen M.L., Louie S.G. (2012). BerkeleyGW: A massively parallel computer package for the calculation of the quasiparticle and optical properties of materials and nanostructures. Comput. Phys. Commun..

[202] Gillet Y., Giantomassi M., Gonze X. (2016). Efficient on-the-fly interpolation technique for Bethe–Salpeter calculations of optical spectra. Comput. Phys. Commun..

[203] Sangalli D., Ferretti A., Miranda H., Attaccalite C., Marri I., Cannuccia E., Melo P., Marsili M., Paleari F., Marrazzo A. (2019). Many-body perturbation theory calculations using the yambo code. J. Phys. Condens. Matter.

[204] Ono S., Noguchi Y., Sahara R., Kawazoe Y., Ohno K. (2015). TOMBO: All-electron mixed-basis approach to condensed matter physics. Comput. Phys. Commun..

[205] Vorwerk C., Aurich B., Cocchi C., Draxl C. (2019). Bethe–Salpeter equation for absorption and scattering spectroscopy: Implementation in the exciting code. Electron. Struct..

[206] Sander T., Maggio E., Kresse G. (2015). Beyond the Tamm-Dancoff approximation for extended systems using exact diagonalization. Phys. Rev. B.

[207] Marini A., Hogan C., Grüning M., Varsano D. (2009). Yambo: An ab initio tool for excited state calculations. Comput. Phys. Commun..

[208] Giannozzi P., Andreussi O., Brumme T., Bunau O., Nardelli M.B., Calandra M., Car R., Cavazzoni C., Ceresoli D., Cococcioni M. (2017). Advanced capabilities for materials modelling with Quantum ESPRESSO. J. Phys. Condens. Matter.

[209] Cheiwchanchamnangij T., Lambrecht W.R.L. (2012). Quasiparticle band structure calculation of monolayer, bilayer, and bulk MoS_2_. Phys. Rev. B.

[210] Komsa H.P., Krasheninnikov A.V. (2012). Effects of confinement and environment on the electronic structure and exciton binding energy of MoS_2_ from first principles. Phys. Rev. B.

[211] Qiu D.Y., da Jornada F.H., Louie S.G. (2013). Optical Spectrum of *MoS*_2_: Many-Body Effects and Diversity of Exciton States. Phys. Rev. Lett..

[212] Shi H., Pan H., Zhang Y.W., Yakobson B.I. (2013). Quasiparticle band structures and optical properties of strained monolayer MoS_2_ and WS_2_. Phys. Rev. B.

[213] Bernardi M., Palummo M., Grossman J.C. (2013). Extraordinary Sunlight Absorption and One Nanometer Thick Photovoltaics Using Two-Dimensional Monolayer Materials. Nano Lett..

[214] Conley H.J., Wang B., Ziegler J.I., Haglund R.F., Pantelides S.T., Bolotin K.I. (2013). Bandgap Engineering of Strained Monolayer and Bilayer MoS_2_. Nano Lett..

[215] Klots A.R., Newaz A.K.M., Wang B., Prasai D., Krzyzanowska H., Lin J., Caudel D., Ghimire N.J., Yan J., Ivanov B.L. (2014). Probing excitonic states in suspended two-dimensional semiconductors byphotocurrent spectroscopy. Sci. Rep..

[216] Soklaski R., Liang Y., Yang L. (2014). Temperature effect on optical spectra of monolayer molybdenum disulfide. Appl. Phys. Lett..

[217] Ugeda M.M., Bradley A.J., Shi S.F., da Jornada F.H., Zhang Y., Qiu D.Y., Ruan W., Mo S.K., Hussain Z., Shen Z.X. (2014). Giant bandgap renormalization and excitonic effects in a monolayer transition metal dichalcogenide semiconductor. Nat. Mater..

[218] Yu H., Liu G.B., Gong P., Xu X., Yao W. (2014). Dirac cones and Dirac saddle points of bright excitons in monolayer transition metal dichalcogenides. Nat. Commun..

[219] Qiu D.Y., Cao T., Louie S.G. (2015). Nonanalyticity, Valley Quantum Phases, and Lightlike Exciton Dispersion in Monolayer Transition Metal Dichalcogenides: Theory and First-Principles Calculations. Phys. Rev. Lett..

[220] Latini S., Olsen T., Thygesen K.S. (2015). Excitons in van der Waals heterostructures: The important role of dielectric screening. Phys. Rev. B.

[221] Qiu D.Y., da Jornada F.H., Louie S.G. (2016). Screening and many-body effects in two-dimensional crystals: Monolayer *MoS*_2_. Phys. Rev. B.

[222] Molina-Sánchez A., Palummo M., Marini A., Wirtz L. (2016). Temperature-dependent excitonic effects in the optical properties of single-layer *MoS*_2_. Phys. Rev. B.

[223] Robert C., Picard R., Lagarde D., Wang G., Echeverry J.P., Cadiz F., Renucci P., Högele A., Amand T., Marie X. (2016). Excitonic properties of semiconducting monolayer and bilayer *MoT*e_2_. Phys. Rev. B.

[224] Despoja V., Rukelj Z., Marušić L. (2016). Ab initio study of electronic excitations and the dielectric function in molybdenum disulfide monolayer. Phys. Rev. B.

[225] Guo L., Wu M., Cao T., Monahan D.M., Lee Y.H., Louie S.G., Fleming G.R. (2019). Exchange-driven intravalley mixing of excitons in monolayer transition metal dichalcogenides. Nat. Phys..

[226] Molina-Sánchez A., Sangalli D., Hummer K., Marini A., Wirtz L. (2013). Effect of spin-orbit interaction on the optical spectra of single-layer, double-layer, and bulk MoS_2_. Phys. Rev. B.

[227] Echeverry J.P., Urbaszek B., Amand T., Marie X., Gerber I.C. (2016). Splitting between bright and dark excitons in transition metal dichalcogenide monolayers. Phys. Rev. B.

[228] Yu H., Laurien M., Hu Z., Rubel O. (2019). Exploration of the bright and dark exciton landscape and fine structure of MoS_2_ using G_0_W_0_-BSE. Phys. Rev. B.

[229] Druppel M., Deilmann T., Kruger P., Rohlfing M. (2017). Diversity of trion states and substrate effects in the optical properties of an MoS_2_ monolayer. Nat. Commun..

[230] Torche A., Bester G. (2019). First-principles many-body theory for charged and neutral excitations: Trion fine structure splitting in transition metal dichalcogenides. Phys. Rev. B.

[231] Zhumagulov Y.V., Vagov A., Senkevich N.Y., Gulevich D.R., Perebeinos V. (2020). Three-particle states and brightening of intervalley excitons in a doped *MoS*_2_ monolayer. Phys. Rev. B.

[232] Zibouche N., Schlipf M., Giustino F. (2021). GW band structure of monolayer *MoS*_2_ using the SternheimerGW method and effect of dielectric environment. Phys. Rev. B.

[233] He J., Hummer K., Franchini C. (2014). Stacking effects on the electronic and optical properties of bilayer transition metal dichalcogenides *MoS*_2_, *MoSe*_2_, *WS*_2_, and *WSe*_2_. Phys. Rev. B.

[234] Gao S., Yang L., Spataru C.D. (2017). Interlayer coupling and gate-tunable excitons in transition metal dichalcogenide heterostructures. Nano Lett..

[235] Gillen R., Maultzsch J. (2018). Interlayer excitons in MoSe_2_/WSe_2_ heterostructures from first principles. Phys. Rev. B.

[236] Torun E., Miranda H.P., Molina-Sánchez A., Wirtz L. (2018). Interlayer and intralayer excitons in MoS_2_/WS_2_ and MoSe_2_/WSe_2_ heterobilayers. Phys. Rev. B.

[237] Lu X., Li X., Yang L. (2019). Modulated interlayer exciton properties in a two-dimensional moiré crystal. Phys. Rev. B.

[238] Gerber I.C., Courtade E., Shree S., Robert C., Taniguchi T., Watanabe K., Balocchi A., Renucci P., Lagarde D., Marie X. (2019). Interlayer excitons in bilayer MoS_2_ with strong oscillator strength up to room temperature. Phys. Rev. B.

[239] Guo H., Zhang X., Lu G. (2020). Shedding light on moiré excitons: A first-principles perspective. Sci. Adv..

[240] Suzuki Y., Watanabe K. (2020). Excitons in two-dimensional atomic layer materials from time-dependent density functional theory: Mono-layer and bi-layer hexagonal boron nitride and transition-metal dichalcogenides. Phys. Chem. Chem. Phys..

[241] Li Z., Lu X., Cordovilla Leon D.F., Lyu Z., Xie H., Hou J., Lu Y., Guo X., Kaczmarek A., Taniguchi T. (2021). Interlayer Exciton Transport in MoSe_2_/WSe_2_ Heterostructures. ACS Nano.

[242] Deilmann T., Rohlfing M., Wurstbauer U. (2020). Light–matter interaction in van der Waals hetero-structures. J. Phys. Condens. Matter.

[243] Palummo M., Bernardi M., Grossman J.C. (2015). Exciton radiative lifetimes in two-dimensional transition metal dichalcogenides. Nano Lett..

[244] Steinhoff A., Rösner M., Jahnke F., Wehling T.O., Gies C. (2014). Influence of Excited Carriers on the Optical and Electronic Properties of MoS_2_. Nano Lett..

[245] Berghäuser G., Malic E. (2014). Analytical approach to excitonic properties of MoS_2_. Phys. Rev. B.

[246] Konabe S., Okada S. (2014). Effect of Coulomb interactions on optical properties of monolayer transition-metal dichalcogenides. Phys. Rev. B.

[247] Wu F., Qu F., MacDonald A.H. (2015). Exciton band structure of monolayer *MoS*_2_. Phys. Rev. B.

[248] Srivastava A., Sidler M., Allain A.V., Lembke D.S., Kis A., Imamoğlu A. (2015). Optically active quantum dots in monolayer WSe_2_. Nat. Nanotechnol..

[249] Scharf B., Frank T., Gmitra M., Fabian J., Žutić I., Perebeinos V. (2016). Excitonic Stark effect in *MoS*_2_ monolayers. Phys. Rev. B.

[250] Ridolfi E., Lewenkopf C.H., Pereira V.M. (2018). Excitonic structure of the optical conductivity in *MoS*_2_ monolayers. Phys. Rev. B.

[251] Steinhoff A., Kim J.H., Jahnke F., Rösner M., Kim D.S., Lee C., Han G.H., Jeong M.S., Wehling T.O., Gies C. (2015). Efficient Excitonic Photoluminescence in Direct and Indirect Band Gap Monolayer MoS_2_. Nano Lett..

[252] Glazov M.M., Amand T., Marie X., Lagarde D., Bouet L., Urbaszek B. (2014). Exciton fine structure and spin decoherence in monolayers of transition metal dichalcogenides. Phys. Rev. B.

[253] Zhou J., Shan W.Y., Yao W., Xiao D. (2015). Berry Phase Modification to the Energy Spectrum of Excitons. Phys. Rev. Lett..

[254] Glazov M.M., Ivchenko E.L., Wang G., Amand T., Marie X., Urbaszek B., Liu B.L. (2015). Spin and valley dynamics of excitons in transition metal dichalcogenide monolayers. Phys. Status Solidi (B).

[255] Van der Donck M., Peeters F.M. (2019). Spectrum of exciton states in monolayer transition metal dichalcogenides: Angular momentum and Landau levels. Phys. Rev. B.

[256] Chaves A.J., Ribeiro R.M., Frederico T., Peres N.M.R. (2017). Excitonic effects in the optical properties of 2D materials: An equation of motion approach. 2D Mater..

[257] Trushin M., Goerbig M.O., Belzig W. (2016). Optical absorption by Dirac excitons in single-layer transition-metal dichalcogenides. Phys. Rev. B.

[258] Glazov M.M., Golub L.E., Wang G., Marie X., Amand T., Urbaszek B. (2017). Intrinsic exciton-state mixing and nonlinear optical properties in transition metal dichalcogenide monolayers. Phys. Rev. B.

[259] Trushin M., Goerbig M.O., Belzig W. (2018). Model Prediction of Self-Rotating Excitons in Two-Dimensional Transition-Metal Dichalcogenides. Phys. Rev. Lett..

[260] Berkelbach T.C., Hybertsen M.S., Reichman D.R. (2013). Theory of neutral and charged excitons in monolayer transition metal dichalcogenides. Phys. Rev. B.

[261] Kylänpää I., Komsa H.P. (2015). Binding energies of exciton complexes in transition metal dichalcogenide monolayers and effect of dielectric environment. Phys. Rev. B.

[262] Mayers M.Z., Berkelbach T.C., Hybertsen M.S., Reichman D.R. (2015). Binding energies and spatial structures of small carrier complexes in monolayer transition-metal dichalcogenides via diffusion Monte Carlo. Phys. Rev. B.

[263] Velizhanin K.A., Saxena A. (2015). Excitonic effects in two-dimensional semiconductors: Path integral Monte Carlo approach. Phys. Rev. B.

[264] Olsen T., Latini S., Rasmussen F., Thygesen K.S. (2016). Simple Screened Hydrogen Model of Excitons in Two-Dimensional Materials. Phys. Rev. Lett..

[265] Kidd D.W., Zhang D.K., Varga K. (2016). Binding energies and structures of two-dimensional excitonic complexes in transition metal dichalcogenides. Phys. Rev. B.

[266] Pedersen T.G. (2016). Exciton Stark shift and electroabsorption in monolayer transition-metal dichalcogenides. Phys. Rev. B.

[267] Cho Y., Berkelbach T.C. (2018). Environmentally sensitive theory of electronic and optical transitions in atomically thin semiconductors. Phys. Rev. B.

[268] Selig M., Berghäuser G., Richter M., Bratschitsch R., Knorr A., Malic E. (2018). Dark and bright exciton formation, thermalization, and photoluminescence in monolayer transition metal dichalcogenides. 2D Mater..

[269] Cavalcante L.S.R., da Costa D.R., Farias G.A., Reichman D.R., Chaves A. (2018). Stark shift of excitons and trions in two-dimensional materials. Phys. Rev. B.

[270] Feierabend M., Khatibi Z., Berghäuser G., Malic E. (2019). Dark exciton based strain sensing in tungsten-based transition metal dichalcogenides. Phys. Rev. B.

[271] Hsu W.T., Quan J., Wang C.Y., Lu L.S., Campbell M., Chang W.H., Li L.J., Li X., Shih C.K. (2019). Dielectric impact on exciton binding energy and quasiparticle bandgap in monolayer WS_2_ and WSe_2_. 2D Mater..

[272] Rytova N.S. (1967). Screened potential of a point charge in a thin film. Proc. MSU Phys. Astron..

[273] Keldysh L.V. (1979). Coulomb interaction in thin semiconduc-tor and semimetal films. JETP Lett..

[274] Slobodeniuk A.O., Bala Ł., Koperski M., Molas M.R., Kossacki P., Nogajewski K., Bartos M., Watanabe K., Taniguchi T., Faugeras C. (2019). Fine structure of K-excitons in multilayers of transition metal dichalcogenides. 2D Mater..

[275] Arora A., Koperski M., Slobodeniuk A., Nogajewski K., Schmidt R., Schneider R., Molas M.R., de Vasconcellos S.M., Bratschitsch R., Potemski M. (2018). Zeeman spectroscopy of excitons and hybridization of electronic states in few-layer WSe_2_, MoSe_2_ and MoTe_2_. 2D Mater..

[276] Das S., Gupta G., Majumdar K. (2019). Layer degree of freedom for excitons in transition metal dichalcogenides. Phys. Rev. B.

[277] Berman O.L., Kezerashvili R.Y. (2016). High-temperature superfluidity of the two-component Bose gas in a transition metal dichalcogenide bilayer. Phys. Rev. B.

[278] Van der Donck M., Peeters F. (2018). Interlayer excitons in transition metal dichalcogenide heterostructures. Phys. Rev. B.

[279] Kormányos A., Zólyomi V., Fal’ko V.I., Burkard G. (2018). Tunable Berry curvature and valley and spin Hall effect in bilayer MoS_2_. Phys. Rev. B.

[280] Berman O.L., Kezerashvili R.Y. (2017). Superfluidity of dipolar excitons in a transition metal dichalcogenide double layer. Phys. Rev. B.

[281] Bieniek M., Szulakowska L., Hawrylak P. (2020). Band nesting and exciton spectrum in monolayer MoS_2_. Phys. Rev. B.

[282] Bieniek M. (2021). Electronic and Optical Properties of Two-Dimensional Transition Metal Dichalcogenide Crystals. Ph.D. Thesis.

[283] Clementi E., Raimondi D.L. (1963). Atomic Screening Constants from SCF Functions. J. Chem. Phys..

[284] Clementi E., Raimondi D.L., Reinhardt W.P. (1967). Atomic Screening Constants from SCF Functions. II. Atoms with 37 to 86 Electrons. J. Chem. Phys..

[285] Cappelluti E., Roldan R., Silva-Guillen J.A., Ordejon P., Guinea F. (2013). Tight-binding model and direct-gap/indirect-gap transition in single-layer and multilayer MoS_2_. Phys. Rev. B.

[286] Liu G.B., Shan W.Y., Yao Y., Yao W., Xiao D. (2013). Three-band tight-binding model for monolayers of group-VIB transition metal dichalcogenides. Phys. Rev. B.

[287] Rostami H., Moghaddam A.G., Asgari R. (2013). Effective lattice Hamiltonian for monolayer MoS_2_: Tailoring electronic structure with perpendicular electric and magnetic fields. Phys. Rev. B.

[288] Zahid F., Liu L., Zhu Y., Wang J., Guo H. (2013). A generic tight-binding model for monolayer, bilayer and bulk MoS_2_. AIP Adv..

[289] Fang S., Kuate Defo R., Shirodkar S.N., Lieu S., Tritsaris G.A., Kaxiras E. (2015). Ab initio tight-binding Hamiltonian for transition metal dichalcogenides. Phys. Rev. B.

[290] Ho Y.H., Wang Y.H., Chen H.Y. (2014). Magnetoelectronic and optical properties of a MoS_2_ monolayer. Phys. Rev. B.

[291] Liu G.B., Xiao D., Yao Y., Xu X., Yao W. (2015). Electronic structures and theoretical modelling of two-dimensional group-VIB transition metal dichalcogenides. Chem. Soc. Rev..

[292] Ridolfi E., Le D., Rahman T.S., Mucciolo E.R., Lewenkopf C.H. (2015). A tight-binding model for MoS_2_ monolayers. J. Phys. Condens. Matter.

[293] Shanavas K.V., Satpathy S. (2015). Effective tight-binding model for MX_2_ under electric and magnetic fields. Phys. Rev. B.

[294] Silva-Guillen J.A., San-Jose P., Roldan R. (2016). Electronic Band Structure of Transition Metal Dichalcogenides from Ab Initio and Slater—Koster Tight-Binding Model. Appl. Sci..

[295] Pearce A.J., Mariani E., Burkard G. (2016). Tight-binding approach to strain and curvature in monolayer transition-metal dichalcogenides. Phys. Rev. B.

[296] Slater J.C., Koster G.F. (1954). Simplified LCAO Method for the Periodic Potential Problem. Phys. Rev..

[297] Koskinen P., Mäkinen V. (2009). Density-functional tight-binding for beginners. Comput. Mater. Sci..

[298] Urban A., Reese M., Mrovec M., Elsässer C., Meyer B. (2011). Parameterization of tight-binding models from density functional theory calculations. Phys. Rev. B.

[299] Kormányos A., Zólyomi V., Drummond N.D., Rakyta P., Burkard G., Fal’ko V.I. (2013). Monolayer MoS_2_: Trigonal warping, the *Γ* valley, and spin-orbit coupling effects. Phys. Rev. B.

[300] Kormányos A., Burkard G., Gmitra M., Fabian J., Zólyomi V., Drummond N.D., Fal’ko V. (2015). Kp theory for two-dimensional transition metal dichalcogenide semiconductors. 2D Mater..

[301] Pedersen T.G., Pedersen K., Brun Kriestensen T. (2001). Optical matrix elements in tight-binding calculations. Phys. Rev. B.

[302] Zhang X., Shan W.Y., Xiao D. (2018). Optical Selection Rule of Excitons in Gapped Chiral Fermion Systems. Phys. Rev. Lett..

[303] Cao T., Wu M., Louie S.G. (2018). Unifying Optical Selection Rules for Excitons in Two Dimensions: Band Topology and Winding Numbers. Phys. Rev. Lett..

[304] Szabo A., Ostlund N.S. (1989). Modern Quantum Chemistry: Introduction to Advanced Electronic Structure Theory.

[305] Shavitt I., Bartlett R.J. (2009). Many-Body Methods in Chemistry and Physics: MBPT and Coupled-Cluster Theory.

[306] Hawrylak P. (1993). Single-electron capacitance spectroscopy of few-electron artificial atoms in a magnetic field: Theory and experiment. Phys. Rev. Lett..

[307] Hawrylak P., Pfannkuche D. (1993). Magnetoluminescence from correlated electrons in quantum dots. Phys. Rev. Lett..

[308] Wojs A., Hawrylak P. (1995). Negatively charged magnetoexcitons in quantum dots. Phys. Rev. B.

[309] Wojs A., Hawrylak P., Fafard S., Jacak L. (1996). Electronic structure and magneto-optics of self-assembled quantum dots. Phys. Rev. B.

[310] Wojs A., Hawrylak P. (1997). Theory of photoluminescence from modulation-doped self-assembled quantum dots in a magnetic field. Phys. Rev. B.

[311] Hawrylak P., Narvaez G.A., Bayer M., Forchel A. (2000). Excitonic Absorption in a Quantum Dot. Phys. Rev. Lett..

[312] Güçlü A.D., Potasz P., Voznyy O., Korkusinski M., Hawrylak P. (2009). Magnetism and Correlations in Fractionally Filled Degenerate Shells of Graphene Quantum Dots. Phys. Rev. Lett..

[313] Zieliński M., Korkusiński M., Hawrylak P. (2010). Atomistic tight-binding theory of multiexciton complexes in a self-assembled InAs quantum dot. Phys. Rev. B.

[314] Güçlü A.D., Potasz P., Hawrylak P. (2010). Excitonic absorption in gate-controlled graphene quantum dots. Phys. Rev. B.

[315] Bayer M., Stern O., Hawrylak P., Fafard S., Forchel A. (2000). Hidden symmetries in the energy levels of excitonic ‘artificial atoms’. Nature.

[316] Korkusinski M., Voznyy O., Hawrylak P. (2011). Theory of highly excited semiconductor nanostructures including Auger coupling: Exciton-biexciton mixing in CdSe nanocrystals. Phys. Rev. B.

[317] Potasz P., Güçlü A.D., Wójs A., Hawrylak P. (2012). Electronic properties of gated triangular graphene quantum dots: Magnetism, correlations, and geometrical effects. Phys. Rev. B.

[318] Korkusinski M., Hawrylak P. (2013). Atomistic theory of emission from dark excitons in self-assembled quantum dots. Phys. Rev. B.

[319] Ozfidan I., Korkusinski M., Hawrylak P. (2015). Theory of biexcitons and biexciton-exciton cascade in graphene quantum dots. Phys. Rev. B.

[320] Güçlü A.D., Potasz P., Korkusinski M., Hawrylak P. (2014). Graphene Quantum Dots.

[321] Fan F., Voznyy O., Sabatini R.P., Bicanic K.T., Adachi M.M., McBride J.R., Reid K.R., Park Y.S., Li X., Jain A. (2017). Continuous-wave lasing in colloidal quantum dot solids enabled by facet-selective epitaxy. Nature.

[322] Cygorek M., Otten M., Korkusinski M., Hawrylak P. (2020). Accurate and efficient description of interacting carriers in quantum nanostructures by selected configuration interaction and perturbation theory. Phys. Rev. B.

[323] Wójs A., Quinn J.J., Hawrylak P. (2000). Charged excitons in a dilute two-dimensional electron gas in a high magnetic field. Phys. Rev. B.

[324] Wójs A., Quinn J.J. (2000). Photoluminescence from fractional quantum Hall systems: Role of separation between electron and hole layers. Phys. Rev. B.

[325] Benedict L.X. (2002). Screening in the exchange term of the electron-hole interaction of the Bethe-Salpeter equation. Phys. Rev. B.

[326] Qiu D.Y., da Jornada F.H., Louie S.G. (2021). Solving the Bethe-Salpeter equation on a subspace: Approximations and consequences for low-dimensional materials. Phys. Rev. B.

[327] Sauer M.O., Nielsen C.E.M., Merring-Mikkelsen L., Pedersen T.G. (2021). Optical emission from light-like and particle-like excitons in monolayer transition metal dichalcogenides. Phys. Rev. B.

[328] Ozfidan I., Korkusinski M., Güçlü A.D., McGuire J.A., Hawrylak P. (2014). Microscopic theory of the optical properties of colloidal graphene quantum dots. Phys. Rev. B.

[329] Sundararaman R., Arias T.A. (2013). Regularization of the Coulomb singularity in exact exchange by Wigner-Seitz truncated interactions: Towards chemical accuracy in nontrivial systems. Phys. Rev. B.

[330] Trolle M.L., Seifert G., Pedersen T.G. (2014). Theory of excitonic second-harmonic generation in monolayer *MoS*_2_. Phys. Rev. B.

[331] Cudazzo P., Tokatly I.V., Rubio A. (2011). Dielectric screening in two-dimensional insulators: Implications for excitonic and impurity states in graphane. Phys. Rev. B.

[332] Quintela M.F.M., Peres N.M. (2020). A colloquium on the variational method applied to excitons in 2D materials. Eur. Phys. J. B.

[333] Tamulewicz M., Kutrowska-Girzycka J., Gajewski K., Serafińczuk J., Sierakowski A., Jadczak J., Bryja L., Gotszalk T.P. (2019). Layer number dependence of the work function and optical properties of single and few layers MoS_2_: Effect of substrate. Nanotechnology.

[334] Berkelbach T.C., Reichman D.R. (2018). Optical and Excitonic Properties of Atomically Thin Transition-Metal Dichalcogenides. Annu. Rev. Condens. Matter Phys..

[335] Ovesen S., Brem S., Linderälv C., Kuisma M., Korn T., Erhart P., Selig M., Malic E. (2019). Interlayer exciton dynamics in van der Waals heterostructures. Commun. Phys..

[336] Kamban H.C., Pedersen T.G. (2020). Interlayer excitons in van der Waals heterostructures: Binding energy, Stark shift, and field-induced dissociation. Sci. Rep..

[337] Kezerashvili R.Y., Spiridonova A. (2021). Magnetoexcitons in transition metal dichalcogenides monolayers, bilayers, and van der Waals heterostructures. Phys. Rev. Res..

[338] Garate I., Franz M. (2011). Excitons and optical absorption on the surface of a strong topological insulator with a magnetic energy gap. Phys. Rev. B.

[339] Srivastava A., Imamoglu A. (2015). Signatures of Bloch-Band Geometry on Excitons: Nonhydrogenic Spectra in Transition-Metal Dichalcogenides. Phys. Rev. Lett..

[340] Sadecka K. (2022). Inter- and Intralayer Excitonic Spectrum of MoSe_2_/WSe_2_ Heterostructure. Acta Phys. Pol. A.

